# Insulinopathies of the brain? Genetic overlap between somatic insulin-related and neuropsychiatric disorders

**DOI:** 10.1038/s41398-022-01817-0

**Published:** 2022-02-14

**Authors:** Giuseppe Fanelli, Barbara Franke, Ward De Witte, I. Hyun Ruisch, Jan Haavik, Veerle van Gils, Willemijn J. Jansen, Stephanie J. B. Vos, Lars Lind, Jan K. Buitelaar, Tobias Banaschewski, Søren Dalsgaard, Alessandro Serretti, Nina Roth Mota, Geert Poelmans, Janita Bralten

**Affiliations:** 1 Department of Biomedical and Neuromotor Sciences, University of Bologna, Bologna, Italy; 2 Department of Human Genetics, Radboud University Medical Center, Donders Institute for Brain, Cognition and Behaviour, Nijmegen, The Netherlands; 3 Department of Psychiatry, Radboud University Medical Center, Donders Institute for Brain, Cognition and Behaviour, Nijmegen, The Netherlands; 4 Department of Child and Adolescent Psychiatry, University of Groningen, University Medical Center Groningen, Groningen, The Netherlands; 5 Department of Biomedicine, University of Bergen, Bergen, Norway; 6 Division of Psychiatry, Haukeland University Hospital, Bergen, Norway; 7 Department of Psychiatry and Neuropsychology, School for Mental Health and Neuroscience, Maastricht University, Maastricht, The Netherlands; 8 Department of Medical Sciences, Uppsala University, Uppsala, Sweden; 9 Department of Cognitive Neuroscience, Donders Institute for Brain, Cognition and Behaviour, Radboud University Medical Center, Nijmegen, The Netherlands; 10 Department of Child and Adolescent Psychiatry and Psychotherapy, Central Institute of Mental Health, Medical Faculty Mannheim, Heidelberg University, Mannheim, Germany; 11 National Centre for Register-Based Research, Aarhus University, Aarhus, Denmark; 12 The Lundbeck Foundation Initiative for Integrative Psychiatric Research, PSYCH, Aarhus, Denmark

**Keywords:** Genomics, Clinical genetics, Psychiatric disorders

## Abstract

The prevalence of somatic insulinopathies, like metabolic syndrome (MetS), obesity, and type 2 diabetes mellitus (T2DM), is higher in Alzheimer’s disease (AD), autism spectrum disorder (ASD), and obsessive-compulsive disorder (OCD). Dysregulation of insulin signalling has been implicated in these neuropsychiatric disorders, and shared genetic factors might partly underlie this observed multimorbidity. We investigated the genetic overlap between AD, ASD, and OCD with MetS, obesity, and T2DM by estimating pairwise global genetic correlations using the summary statistics of the largest available genome-wide association studies for these phenotypes. Having tested these hypotheses, other potential brain “insulinopathies” were also explored by estimating the genetic relationship of six additional neuropsychiatric disorders with nine insulin-related diseases/traits. Stratified covariance analyses were then performed to investigate the contribution of insulin-related gene sets. Significant negative genetic correlations were found between OCD and MetS (*r*_g_ = −0.315, *p* = 3.9 × 10^−8^), OCD and obesity (*r*_g_ = −0.379, *p* = 3.4 × 10^−5^), and OCD and T2DM (*r*_g_ = −0.172, *p* = 3 × 10^−4^). Significant genetic correlations with insulin-related phenotypes were also found for anorexia nervosa (AN), attention-deficit/hyperactivity disorder (ADHD), major depressive disorder, and schizophrenia (*p* < 6.17 × 10^−4^). Stratified analyses showed negative genetic covariances between AD, ASD, OCD, ADHD, AN, bipolar disorder, schizophrenia and somatic insulinopathies through gene sets related to insulin signalling and insulin receptor recycling, and positive genetic covariances between AN and T2DM, as well as ADHD and MetS through gene sets related to insulin processing/secretion (*p* < 2.06 × 10^−4^). Overall, our findings suggest the existence of two clusters of neuropsychiatric disorders, in which the genetics of insulin-related diseases/traits may exert divergent pleiotropic effects. These results represent a starting point for a new research line on “insulinopathies” of the brain.

## Introduction

Mental disorders are characterised by a reduced life expectancy of ~10 years [1]. In addition to violent causes of death, more than 67% of the increase in premature mortality is due to natural causes [2]. The increased prevalence of insulin-related somatic diseases (i.e., type 2 diabetes mellitus (T2DM), obesity, and metabolic syndrome (MetS)) observed in mental disorders, with a resulting increased cardiovascular risk, contributes significantly to the lower life expectancy [3].

A number of studies have investigated this higher comorbidity, focusing mainly on metabolic disturbances as possible consequences of unhealthy lifestyles, sedentary habits, or the chronic use of psychotropic medication [4]. However, there is growing evidence for the presence of glycaemic and metabolic imbalances in drug-naïve acute psychiatric patients already at disease onset, suggesting that common pathogenic mechanisms may also be involved [5]. Shared genetic factors may play a role, and genomic studies may help to unravel the biological underpinnings of the phenotypically observed comorbidity of neuropsychiatric disorders with somatic insulin-related diseases and traits.

The above-mentioned insulin-related and neuropsychiatric diagnostic groups consist of complex and heterogeneous diseases with a highly polygenic inheritance pattern; heritability estimates from twin and family studies range between 30% and 80% [6, 7]. Large meta-analyses of genome-wide association studies (GWASs) have identified hundreds of disease-associated single nucleotide polymorphisms (SNPs), each contributing with a small effect to the overall risk for these diseases [8]. Genetic sharing has already been highlighted between T2DM, obesity and MetS, as expected from their highly interrelated pathogenesis [9], and recent evidence has also revealed the presence of substantial pleiotropy among psychiatric disorders [10].

A key feature that T2DM, obesity and MetS have in common is an impaired response to insulin stimulation in peripheral tissues, better known as insulin resistance [11]. Abnormalities in insulin signalling might also link with neuropsychiatric disorders. Indeed, beyond the anabolic function of insulin at the peripheral level, where it promotes glucose uptake in tissues while stimulating glycogenesis and lipogenesis, this hormone can also bind to insulin receptors (INSRs) on the surface of both neurons and glial cells in the central nervous system (CNS) [11], where insulin signalling is regulated a.o. by the neurotransmitters serotonin and dopamine [12]. In the CNS, insulin plays a key role in synaptic plasticity and neurotransmission, apoptosis inhibition, and neuroinflammation [13]. Preclinical studies have suggested that an increase in the mammalian target of rapamycin (mTOR) activity, one of the major downstream effectors of the INSRs, may lead to reduced synaptic pruning, and thereby contributes to the cognitive inflexibility and perseverative/repetitive behaviours observed in those animals with *mTOR* genetic alterations [14, 15]. Cognitive abnormalities of a similar nature were shown in TALLYHO/JngJ mice, an animal model of T2DM [16].

Recently, dysregulation in insulin signalling has been suggested to contribute to neuropsychiatric disorders more widely. Evidence is strongest for Alzheimer’s disease (AD) and autism spectrum disorder (ASD) [17–22]. Our own recent work also suggested a link with obsessive-compulsive disorder (OCD) [18, 22]. In the case of AD, it has been shown that insulin sensitivity is altered even before the onset of cognitive decline or β-amyloid (Aβ) accumulation in the CNS [20]. The hyperactivity of the phosphatidylinositol-3-kinase (PI3K)/protein kinase B (AKT)/mTOR cascade, mediated by the phosphorylation of INSR via insulin binding to the neuronal surface, leads to the inhibition of autophagy processes and subsequent accumulation of damaged mitochondria and misfolded proteins seen in AD [19]. The same PI3K/AKT/mTOR hyperactivity is also involved in ASD pathogenesis [17], and genes within the mTOR pathway were also shown to associate with brain volume variability and ASD [23]. Furthermore, offspring of mothers who have T2DM during pregnancy have a higher risk of developing ASD [21]. The integration of data from different types of genetic studies has also implicated CNS insulin signalling as one of the biological mechanisms underlying OCD, where this signalling pathway may modulate excitatory synaptogenesis and postsynaptic dendritic spine formation [18]. Also, obsessive-compulsive symptoms in the general population have been associated with genes related to CNS insulin signalling [22], and shared genetic aetiologies of peripheral insulin-related phenotypes (i.e., T2DM, glucose levels 2 h after an oral glucose challenge (2hGlu), and fasting plasma insulin (FPI)) were found with both obsessive-compulsive symptoms and OCD [22].

In light of the above evidence, we aimed to investigate the extent of the potential genetic sharing and contribution of insulin-related gene sets in the observed comorbidity of neuropsychiatric disorders having preclinical evidence of insulin signalling dysregulation (i.e., AD, ASD, and OCD) with somatic diseases related to insulin resistance, namely MetS, obesity, and T2DM. For this purpose, we performed Linkage Disequilibrium SCore regression (LDSC) and stratified GeNetic cOVariance Analyzer (GNOVA) analyses [24, 25]. In addition, we explored other potential brain “insulinopathies” by estimating the genetic overlap between other neuropsychiatric disorders and insulin-related somatic phenotypes.

## Materials and methods

### Input datasets

As input for the analyses, we used summary statistic data of the largest GWASs available at the time of conducting our analyses for the phenotypes of interest (see also Table 1 and the Supplementary information). We considered the most prevalent somatic diseases linked to insulin resistance (i.e., MetS, obesity, and T2DM), and neuropsychiatric disorders having preclinical evidence of insulin signalling dysregulation, namely AD, ASD, and OCD [15, 16, 20]. We also investigated insulin-related traits (i.e., 2hGlu, body mass index (BMI), fasting plasma glucose (FPG) and FPI, glycated haemoglobin (HbA1c), and homeostatic model assessment for insulin resistance (HOMA-IR)), and other six neuropsychiatric disorders, which are those best characterised genetically by the Psychiatric Genomics Consortium [10] (i.e., attention-deficit/hyperactivity disorder (ADHD), anorexia nervosa (AN), bipolar disorder (BD), major depressive disorder (MDD), schizophrenia (SCZ), and Tourette’s syndrome (TS)). Data were downloaded from online repositories (see URLs), when publicly available, or requested (i.e., MetS) from the GWAS authors.

**Table 1:** Characteristics of the samples used for the Linkage-Disequilibrium SCore regression (LDSC) and GeNetic cOVariance Analyzer (GNOVA) analyses.

Trait/disorder	Author	Year	PMID	Consortium	Ancestry	N	Cases	Controls	N_eff_
2hGlu	Saxena et al.	2010	20081857	MAGIC	European	15,234			
BMI	Pulit et al.	2019	30239722	GIANT	European	697,734			
FPG	Lagou et al.	2021	33402679	MAGIC	European	140,595			
FPI	Lagou et al.	2021	33402679	MAGIC	European	98,210			
HbA1c	Wheeler et al.	2017	28898252	MAGIC	European	123,665			
HOMA-IR	Dupuis et al.	2010	20081858	MAGIC	European	37,037			
MetS	Lind	2019	31589552		European	291,107	59,677	231,430	189,772.64
Obesity	Watanabe et al.	2019	31427789		European	244,890	9805	235,085	37,649.69
T2DM	Mahajan et al.	2018	30297969	DIAGRAM	European	898,130	74,124	824,006	272,025.75
ADHD	Demontis et al.	2019	30478444	PGC	European	53,293	19,099	34,194	49,017.41
AD	Wightman et al.	2021	34493870	PGC	European	762,917	86,531	676,386	306,866.18
AN	Watson et al.	2019	31308545	PGC	European	72,517	16,992	55,525	52,041.91
ASD	Grove et al.	2019	30804558	PGC	European	46,350	18,381	27,969	44,366.62
BD	Mullins et al.	2021	34002096	PGC	European	413,466	41,917	371,549	150,669.89
OCD	OCGAS/IOCDF-GC	2018	28761083	OCGAS/IOCDF-GC	European	9725	2688	7037	7780.14
MDD	Wray et al./Howard et al.	2019	29700475/ 29662059	PGC	European	500,199	170,756	329,443	449,855.91
SCZ	Pardinas et al.	2018	29483656	PGC + CLOZUK	European	105,318	40,675	64,643	99,863.42
TS	Yu et al.	2019	30818990	PGC	European	14,307	4819	9488	12,783.30

*2hGlu* glucose levels 2 h after an oral glucose challenge, *BMI* body mass index, *FPG* fasting plasma glucose, *FPI* fasting plasma insulin, *HbA1c* glycated haemoglobin, *HOMA-IR* homeostatic model assessment for insulin resistance, *MetS* metabolic syndrome, *T2DM* type 2 diabetes mellitus, *ADHD* attention-deficit/hyperactivity disorder, *AD* Alzheimer’s disease, *AN* anorexia nervosa, *ASD* autism spectrum disorder, *BD* bipolar disorder, *MDD* major depressive disorder, *OCD* obsessive-compulsive disorder, *SCZ* schizophrenia, *TS* Tourette’s syndrome, *N* total sample size, *N*_*eff*_ effective sample size [N_eff_ = 4/(1/Cases +  1/Controls)].

### Genome-wide bivariate genetic correlation estimations

Bivariate LDSC (https://github.com/bulik/ldsc) analyses were performed to estimate the genetic correlation (*r*_g_) ascribed genome-wide to common variants between AD, ASD, OCD and MetS, obesity, and T2DM, following the software guidelines (https://github.com/bulik/ldsc/wiki/Heritability-and-Genetic-Correlation). Also through LDSC, exploratory analyses were carried out to estimate the extent of the genetic sharing between other neuropsychiatric disorders (ADHD, AN, BD, MDD, SCZ, TS, along with AD, ASD, and OCD) and insulin-related somatic diseases/traits (i.e., 2hGlu, BMI, FPG and FPI, HbA1c, HOMA-IR, along with MetS, obesity, and T2DM). Further details on the quality control (QC) steps and the LDSC method are provided in the Supplementary information. LDSC is computationally robust to sample overlaps between studies [24]. Bonferroni correction was applied, accounting for the number of analyses performed (*α* = 0.05/(9 × 9) = 6.17 × 10^−4^).

### Genetic covariance analyses stratified by functional annotations

GNOVA (https://github.com/xtonyjiang/GNOVA) was used to investigate whether neuropsychiatric disorders were genetically correlated to MetS, obesity, or T2DM specifically through nine gene sets involved in peripheral and/or CNS insulin signalling (gene set sizes ranged from 27 to 137 genes; see Tables S1–S2 for a complete list of genes included in each gene set). Further details on the GNOVA method and the selection of the insulin signalling-related gene sets are provided in the Supplementary information. GNOVA-computed covariance estimates are robust to sample overlaps [25]. Bonferroni correction was applied to GNOVA results considering the nine tested gene sets and the 27 pairwise combinations of three insulin-related somatic diseases and nine neuropsychiatric disorders for which GNOVA analyses were performed (*α* = 0.05/(9 × 3 × 9) = 2.06 × 10^−4^).

## Results

### Description of the input datasets

A description of the samples (with sample sizes, number of cases and controls, and the derived effective sample size) included in the analyses is provided in Table 1. Further information on the GWAS samples can be found in the Supplementary information.

### Pairwise genome-wide genetic correlations between neuropsychiatric disorders and insulin-related somatic diseases and traits

**Fig. 1:**
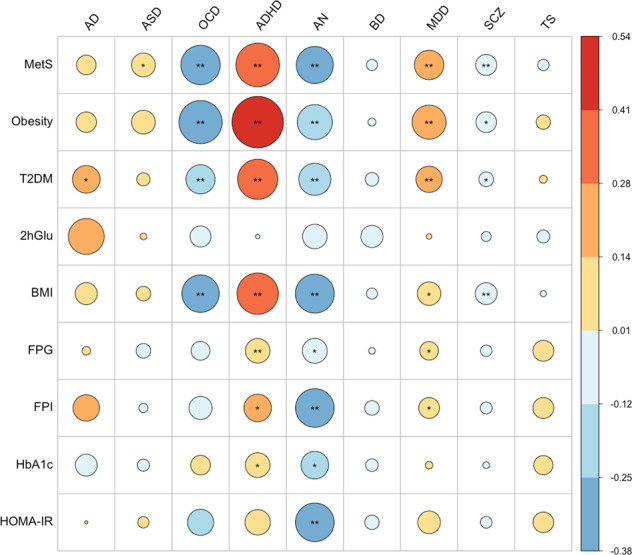
Genetic correlation plot summarising the results from the bivariate Linkage Disequilibrium SCore regression (LDSC) analyses. The size of the circle is proportional to the genetic correlation estimates, going from warmer to colder colours as the direction of the effect changes from positive to negative. Bonferroni multiple testing correction was applied, correcting for the number of analyses performed (*α* = 0.05/(9 x 9) = 6.17e−4). AD Alzheimer’s disease, ASD autism spectrum disorder, OCD obsessive-compulsive disorder, ADHD attention-deficit/hyperactivity disorder, AN anorexia nervosa, BD bipolar disorder, MDD major depressive disorder, SCZ schizophrenia, TS Tourette’s syndrome, MetS metabolic syndrome, T2DM type 2 diabetes mellitus, 2hGlu glucose levels 2 h after an oral glucose challenge, BMI body mass index, FPG fasting plasma glucose, FPI fasting plasma insulin, HbA1c glycated haemoglobin, HOMA-IR homeostatic model assessment for insulin resistance. ** Statistically significant bivariate genetic correlation (*p* < 6.17 × 10^−4^). * Nominally significant bivariate genetic correlation (*p* < 0.05).

A genetic correlation plot depicting the LDSC analyses results is shown in Fig. 1; details on the genetic correlation estimates (*r*_g_) for each pair and statistical significance are provided in Table 2. After correcting for multiple testing, negative genetic correlations were highlighted between OCD and MetS (*r*_g_ = −0.315, *p* = 3.9 × 10^−8^), OCD and obesity (*r*_g_ = −0.379, *p* = 3.6 × 10^−5^), and OCD and T2DM (*r*_g_ = −0.172, *p* = 3 × 10^−4^). Nominally significant genetic correlations were also found between AD and T2DM (*r*_g_ = 0.155, *p* = 0.048), and ASD and MetS (*r*_g_ = 0.115, *p* = 0.002).

**Table 2:** Genetic correlation table reporting the detailed results derived from the bivariate Linkage Disequilibrium SCore regression (LDSC) analyses.

Trait/disorder	AD	ASD	OCD	ADHD	AN	BD	MDD	SCZ	TS
MetS	0.078 (0.239)	0.115 (0.002)*	−0.315 (3.88 × 10^−8^)**	0.386 (7.16 × 10^−30^)**	−0.279 (3.43 × 10^−15^)**	−0.025 (0.321)	0.177 (1.66 × 10^−16^)**	−0.090 (1.41 × 10^−5^)**	−0.026 (0.496)
Obesity	0.085 (0.455)	0.115 (0.072)	−0.379 (3.35 × 10^−5^)**	0.538 (9.91 × 10^−24^)**	−0.250 (7.60 × 10^−6^)**	−0.013 (0.749)	0.235 (5.31 × 10^−10^)**	−0.087 (0.009)*	0.042 (0.552)
T2DM	0.155 (0.048)*	0.035 (0.403)	−0.172 (3 × 10^−4^)**	0.328 (3.24 × 10^−28^)**	−0.209 (6.04 × 10^−12^)**	−0.037 (0.094)	0.141 (4.65 × 10^−11^)**	−0.044 (0.016)*	0.013 (0.713)
2hGlu	0.261 (0.103)	0.009 (0.936)	−0.090 (0.591)	−0.004 (0.964)	−0.122 (0.221)	−0.100 (0.180)	0.006 (0.927)	−0.020 (0.743)	−0.034 (0.782)
BMI	0.099 (0.126)	0.043 (0.164)	−0.284 (2.57 × 10^−11^)**	0.348 (6.59 × 10^−49^)**	−0.308 (6.38 × 10^−38^)**	−0.025 (0.167)	0.112 (1.55 × 10^−10^)**	−0.097 (7.95 × 10^−11^)**	−0.008 (0.801)
FPG	0.015 (0.828)	−0.043 (0.334)	−0.072 (0.339)	0.123 (6 × 10^−4^)**	−0.126 (0.005)*	−0.009 (0.777)	0.070 (0.012)*	−0.027 (0.296)	0.089 (0.074)
FPI	0.142 (0.218)	−0.017 (0.797)	−0.108 (0.198)	0.154 (0.005)*	−0.303 (4.17 × 10^−7^)**	−0.043 (0.319)	0.088 (0.045)*	−0.029 (0.464)	0.089 (0.190)
HbA1C	−0.097 (0.265)	−0.030 (0.619)	0.079 (0.367)	0.124 (0.006)*	−0.155 (0.003)*	−0.032 (0.379)	0.012 (0.722)	−0.009 (0.751)	0.075 (0.225)
HOMA-IR	0.002 (0.985)	0.026 (0.817)	−0.139 (0.255)	0.1313 (0.138)	−0.3029 (1 × 10^−4^)**	−0.042 (0.505)	0.1044 (0.076)	−0.0278 (0.577)	0.086 (0.390)

Reported values are genetic correlation estimates – *r*_g_ – (*p*-values).

*AD* Alzheimer’s disease, *ASD* autism spectrum disorders, *OCD* obsessive-compulsive disorder, *ADHD* attention-deficit/hyperactivity disorder, *AN* anorexia nervosa, *BD* bipolar disorder, *MDD* major depressive disorder, *SCZ* schizophrenia, *TS* Tourette’s syndrome, *MetS* metabolic syndrome, *T2DM* type 2 diabetes mellitus, *2hGlu* glucose levels 2 h after an oral glucose challenge, *BMI* body mass index, *FPG* fasting plasma glucose, *FPI* fasting plasma insulin, *HbA1c* glycated hemoglobin, *HOMA-IR* homeostatic model assessment for insulin resistance.

** Statistically significant bivariate genetic correlation (*p* < 6.17 × 10^−4^).

* Nominally significant bivariate genetic correlation (*p* < 0.05).

When insulin-related somatic traits (i.e., 2hGlu, BMI, FPG, FPI, HbA1c, HOMA-IR) were considered, OCD was also found to be significantly negatively genetically correlated with BMI (*r*_g_ = −0.284, *p* = 2.6 × 10^−11^), but neither AD nor ASD showed significant correlations with the traits.

Analyses were also extended to other neuropsychiatric disorders (i.e., ADHD, AN, BD, MDD, SCZ, and TS) and significant genetic correlations were found between insulin-related diseases/traits and ADHD, AN, MDD, and SCZ (see Fig. 1 and Table 2).

### Genetic covariance between neuropsychiatric disorders and insulin-related somatic diseases stratified by insulin-related gene sets

After Bonferroni correction, stratified GNOVA analyses highlighted significant negative genetic covariance between AD and obesity through the Reactome INSR recycling gene set (*p* = 4.6 × 10^−5^), as well as between ASD and MetS through the Biocarta, KEGG, and PID insulin signalling pathways (*p* ≤ 3.2 × 10^−5^). OCD showed negative genetic covariance with MetS and T2DM through the Reactome INSR recycling gene set (*p* ≤ 1.6 × 10^−4^).

When the other neuropsychiatric disorders were also considered, negative genetic covariance was found between BD and T2DM, BD and MetS, SCZ and MetS through the PID insulin signalling pathway (*p* ≤ 2 × 10^−5^), as well as between AN and T2DM through the Biocarta insulin pathway (*p* = 1.26 × 10^−5^). Moreover, positive genetic covariance was highlighted between AN and T2DM through the Reactome insulin processing gene set (*p* = 3.77 × 10^−5^), as well as between ADHD and MetS through the Reactome regulation of insulin secretion gene set (*p* = 1.18 × 10^−4^) (see Table 3; detailed results are shown in Tables S3–S11).

**Table 3:** Summary results of the genetic covariance analyses between neuropsychiatric disorders and somatic diseases linked with insulin-resistance stratified by insulin signalling gene sets.

Gene-set name	*n* genes/gene set	Base phenotypes	ρ_g_	SE ρ_g_	*p*	*h*^2^_*SNP*_ (1)	*h*^2^_*SNP*_ (2)	Annotated SNPs	Total SNPs
Biocarta insulin pathway	21	AN × T2DM	−0.00042^a^	0.00010	1.26 × 10^−5 b^	0.00078	0.00050	1268	860,288
Biocarta insulin pathway	21	ASD × MetS	−0.00041	0.00010	1.96 × 10^−5^	0.00046	0.00068	1520	968,964
KEGG insulin signaling pathway	137	ASD × MetS	−0.00170	0.00041	3.22 × 10^−5^	0.00261	0.00207	11,334	968,964
PID insulin pathway	44	ASD × MetS	−0.00080	0.00018	1.25 × 10^−5^	0.00012	0.00105	4319	968,964
PID insulin pathway	44	BD × T2DM	−0.00057^a^	0.00013	9.60 × 10^−6 b^	0.00054	0.00121	4575	1,026,853
PID insulin pathway	44	BD × MetS	−0.00076^a^	0.00018	2.03 × 10^−5 b^	0.00054	0.00109	4580	1,027,553
PID insulin pathway	44	SCZ × MetS	−0.00141	0.00032	1.32 × 10^−5^	0.00155	0.00117	4836	1,049,783
Reactome insulin processing	27	AN × T2DM	0.00059^a^	0.00014	3.77 × 10^−5 b^	0.00216	0.00153	2742	860,288
Reactome regulation of insulin secretion	77	ADHD × MetS	0.00174	0.00045	1.18 × 10^−4^	0.00287	0.00156	9850	986,120
Reactome insulin receptor recycling	26	AD × Obesity	−0.00079^a^	0.00019	4.61 × 10^−5 b^	0.00009	−0.00033	2138	942,664
Reactome insulin receptor recycling	26	OCD × MetS	−0.00124	0.00028	7.5 × 10^−6^	0.00316	0.00074	2132	1,019,413
Reactome insulin receptor recycling	26	OCD × T2DM	−0.00100	0.00026	1.6 × 10^−4^	0.00304	0.00128	2130	1,019,648

Results are only reported for phenotype pairs when the stratified genetic covariance estimates were statistically significant after Bonferroni correction (*p* < 2.06 × 10^−4^).

*AD* Alzheimer’s disease, *ADHD* attention-deficit/hyperactivity disorder, *AN* anorexia nervosa, *ASD* autism spectrum disorder, *BD* bipolar disorder, *OCD* obsessive-compulsive disorder, *SCZ* schizophrenia, *MetS* metabolic syndrome, *T2DM* type 2 diabetes mellitus, *SNPs* single nucleotide polymorphisms, *ρ*_*g*_ genetic covariance estimate, *SE ρ*_*g*_ standard error of the estimate of ρ_g,_ *p* *p*-value from the statistical test for genetic covariance, *r*_*g*_ genetic correlation estimate, *h*^*2*^_*SNP*_ *(1)* SNP-based heritability estimate for the first phenotype, *h*^*2*^_*SNP*_ *(2)* SNP-based heritability estimate for the second phenotype.

^a^ρ_g_ corrected: genetic covariance estimates with sample overlap correction.

^b^*p* corrected: *p*-value from the statistical test for genetic covariance with sample overlap correction.

## Discussion

In this study, we investigated the genetic overlap of AD, ASD, OCD with somatic insulinopathies, namely MetS, obesity and T2DM, hypothesising an important role for gene sets related to insulin signalling. Our genome-wide analyses indicate significant global negative genetic correlations between OCD and obesity, T2DM, and MetS. Gene set stratified genetic covariance analyses of specific insulin-related pathways helped identify a genetic link of AD, ASD, and OCD with somatic insulinopathies. Moreover, our exploration of other potential brain “insulinopathies” yielded evidence for global genetic overlap of ADHD, AN, MDD, and SCZ with somatic insulin-related diseases/traits, while genetic covariance at the level of insulin-related gene sets was identified between ADHD, AN, BD, SCZ and T2DM/MetS/obesity.

The previous clinical and epidemiological studies available to date indicate a higher prevalence of obesity, MetS, and T2DM in patients with OCD than the general population [26, 27]. Furthermore, a mouse model for T2DM showed compulsive traits, as discussed above [20]. We thus had hypothesised a genetic correlation between OCD and somatic disorders characterised by insulin resistance to exist, which we indeed found in this study. The negative direction of the correlation we found was unexpected, as it might suggest a protective role of the genetics underlying OCD on the chance of having T2DM, MetS and/or obesity. However, for behavioural traits, environmental sources of variation may operate orthogonally to genetic factors, masking the effect of the genetics at the phenotypic level [28]. Therefore, one hypothesis explaining our result can be that environmental effects act in the opposite direction to genetics, causing an increased risk in the presence of protective genetics and resulting in variability in the phenotypic manifestations over time. Indeed, metabolic complications have been particularly associated with a longer duration of antipsychotics treatment in patients with OCD [26]. It is also reasonable to assume that patients with more severe symptoms, having higher genetic load for OCD, are more likely to develop metabolic side effects of such treatments because they require higher doses and longer therapies, even though they might be genetically more protected against insulin-related/metabolic disturbances. The analyses considering insulin-related glycaemic/anthropometric traits also showed a negative correlation between OCD and BMI. This finding is consistent with previous evidence in smaller samples of a negative genetic relationship with a negative direction between OCD and body fat measures [29]; it also further supports the negative correlation trend that we observed between OCD and somatic insulinopathies. Zooming in through analyses of gene sets related to insulin signalling, we found genes involved in the INSR recycling process involved in the genetic correlation of OCD with both MetS and T2DM. This molecular pathway mediates the recycling of the INSR and reintegration into the plasma membrane. After activation, the INSR-insulin complex is internalised into the cell within an endosome, and insulin is degraded, while INSR is dephosphorylated and reintegrated into the plasma membrane [30]. To our knowledge, this is the first study reporting involvement of the INSR recycling pathway in neuropsychiatric phenotypes. In this respect, it should be noted that endosomal recycling processes are relevant to the functioning of the brain. They are important for synaptic functioning and plasticity (and related glutamatergic neurotransmission) as well as for the maintenance of levels of membrane proteins, more generally [31].

We did not observe significant genome-wide genetic correlations between AD and somatic insulin-related diseases, only nominally significant positive genetic correlations were seen with MetS and T2DM before multiple testing correction. Our results may add support for a predominant influence of environmental and epigenetic factors in the comorbidity observed between AD and somatic insulinopathies, although we cannot exclude the possible existence of patterns of local genetic correlation [32]. It should be noted that ageing is considered the greatest risk factor for AD, and T2DM incidence also increases with ageing [33]. Processes linked to oxidative damage and ageing could trigger the onset of both diseases in a way that is partly independent from genetic effects [19]. Air pollution, smoking, and low physical activity are also important risk factors for broadly defined dementia, and they also contribute to insulin resistance and cerebrovascular disease [33, 34]. The role of epigenetic modulation, including DNA methylation, histone modifications and non-coding RNAs, in the aetiopathogenesis of AD is also well recognised, and this may provide novel avenues for treatment in the upcoming years [35]. A hypothesis is that the clinical heterogeneity of AD may have camouflaged the presence of genetic factors shared with somatic insulinopathies. In this regard, more deeply phenotyped samples might help better investigate the presence of pleiotropic effects in the future [36]. Alternatively or in addition, previous evidence may point to a role for insulin signalling specifically in individuals carrying *APOE* polymorphisms, suggesting that new insights may be derived from stratification of the AD population according to *APOE* genotype. Indeed, oral antidiabetics, such as thiazolidinediones and intranasal insulin have shown differential efficacy in AD depending on the *APOE*-ε4 genotype [37], which is the strongest common genetic risk factor for late-onset AD [38]. Moreover, a previous study has also shown a strong regional genetic correlation between AD and T2DM for the genetic variants mapped to the apolipoprotein-E (*APOE*) locus [39]. However, the absence of genetic correlations at the genome-wide level does not preclude the existence of genetic sharing, as both positive and negative local genetic correlations may occur and potentially cancel each other out when summed at the genome-wide level [40]. In this regard, we demonstrated significant genetic covariance between AD and obesity at the INSR recycling gene set level. Under physiological conditions, INSR is maintained in equilibrium between an internalising and an exposed state at the plasma membrane [41]. Either excessive or insufficient surface INSR can lead to the development of insulin resistance [41]. Our finding is in line with the evidence of an altered cellular distribution of INSRs in AD, resulting in a loss of INSRs at the neuronal membrane, suggesting that alterations in INSR recycling/trafficking are present [42].

A role of metabolic dysregulation in ASD has been previously suggested by the increased risk for ASD and neurodevelopmental delays in the offspring of mothers who have metabolic conditions during pregnancy [43]. Nevertheless, our study did not find ASD to be significantly genetically correlated at the genome-wide level with either MetS, obesity or T2DM, in line with non-significant previous reports using smaller sample sizes [44]. However, the stratification to insulin-specific gene-sets revealed significant localised negative genetic covariance of ASD with MetS through genes within insulin signalling pathways. Although further studies will be needed to disentangle the biological meaning of this finding, we could speculate that the observed pathway-level negative genetic covariance between ASD and MetS might reflect higher complexity of reciprocal regulation between monoamine and insulin signalling at the CNS and peripheral level [12]. What we found at the gene set level may also be consistent with prior findings of enhanced insulin signaling in the brain of a Drosophila model of Fragile X syndrome, which represents the most prevalent hereditary type of intellectual disability and autism [45].

To extend the spectrum of potential brain “insulinopathies”, LDSC analyses were performed considering six other neuropsychiatric disorders and diseases/traits related to insulin resistance. Our analyses identified several additional genetic correlations of the somatic insulin-related diseases with neuropsychiatric disorders; negative genetic correlations were seen between MetS and both AN and schizophrenia, and positive genetic correlations were observed for MetS with both ADHD and MDD. Of note, the diagnosis of MetS is made when at least three out of the following co-occur: high systolic blood pressure, low levels of high-density lipoprotein (HDL), hyperglycaemia, high levels of triglycerides, and/or increased waist circumference [9]. Our findings are consistent with previous evidence of pairwise genetic sharing between lipidemic traits (HDL and triglycerides), waist circumference and AN, ADHD, and/or MDD [8, 46–48]. In line with the negative genetic correlations that we observed between MetS and both AN and schizophrenia, MR studies have previously identified AN and SCZ as causal for decreased fat mass [29]. This finding may suggest a prevalent contribution of environmental factors, such as the use of antipsychotics, unhealthy diet and lifestyle, reduced access to medical care on the epidemiological evidence of an increased risk of MetS, hypertension, and dyslipidaemia in patients with SCZ [49]. We also replicated and updated previous evidence of genetic sharing of ADHD, AN, and MDD with T2DM, as well as of ADHD, AN, MDD, and SCZ with both obesity and BMI [8, 24, 29, 46, 47, 50]. With regard to SCZ and BMI, the negative direction of the genetic correlation corresponds to the previously reported evidence of a negative association of polygenic risk scores for SCZ with BMI [50]. Exploring further the genetic links between these neuropsychiatric disorders and glycaemic traits linked to insulin resistance, we revealed a novel positive correlation between ADHD and FPG, as well as negative bivariate correlations between AN and both FPI and HOMA-IR that replicate and update previous findings [29, 46]. A Mendelian randomisation study had also previously shown that higher levels of FPI have a causal effect in reducing the risk of AN [51].

Interestingly, the local genetic covariance we have highlighted between neuropsychiatric disorders and somatic diseases linked to insulin resistance was in most cases in the negative direction at the level of gene sets related to insulin signalling, except for AN and ADHD. A negative direction means that genetic variability at the level of these gene sets may result in an opposite pleiotropic effect on these two groups of diseases. However, the biological interpretation of these findings does not seem obvious at present and additional investigations at the gene and functional level will be necessary to clarify their biological significance.

This study comes with some strengths and limitations. The major strength is the investigation of the possible specific involvement of insulin-related gene sets at the genomic level for the first time in the phenotypically observed comorbidity between neuropsychiatric disorders and somatic diseases related to insulin resistance. GNOVA provided us with more powerful statistical inference and more accurate genetic covariance estimates than LDSC and helped dissect the shared genetic architecture of the considered complex diseases, while giving us greater insights into the underlying biology. We exploited the largest public GWAS summary statistics (up to 898,130 individuals for T2DM) and used a strict Bonferroni correction to avoid type-1 errors. Our study may be limited by not having considered in our analyses the potential effect of environmental factors and epigenetic mechanisms, which are likely to mediate the relationship between neuropsychiatric and somatic insulinopathies, as well as potential sex effects due to the unavailability of publicly available sex-stratified data for all the traits/disorders tested and the loss of power for some of the phenotypes investigated. Another limitation is the inclusion of European-only datasets in our analyses, which limits the generalisability of our findings. In addition, the composition of insulin-related gene sets, used as functional annotations in our stratified analyses, may be influenced by the current, still incomplete knowledge of the biology and functioning of the pathways to which they refer.

In conclusion, our study revealed the presence of genetic overlap between OCD and insulin-related somatic diseases, with a likely protective effect of the genetics underlying OCD on the chance of having MetS, obesity, and/or T2DM. However, environmental effects, such as psychotropic drug use, or a relatively unhealthy lifestyle, may act in the opposite direction to genetics, causing increased metabolic risk despite protective genetics. We pointed out that other neuropsychiatric disorders, besides OCD, represent potential brain “insulinopathies”. Two distinct clusters of psychiatric disorders have emerged, in which the genetics of insulin-related traits/diseases may exert divergent pleiotropic effects: one consisting of AN, OCD, and SCZ, which showed negative genetic overlap with somatic insulin-related diseases and traits, and the other one comprising ADHD, and MDD, which showed positive genetic overlap with insulin-related diseases and traits. Finally, we demonstrated that insulin-related gene sets may be pleiotropic for neuropsychiatric disorders (i.e., AN, ADHD, ASD, BD, OCD, and SCZ) and somatic insulinopathies, suggesting that the cumulative effect of genetic variability within insulin-related gene sets on the investigated neuropsychiatric disorders except for AN and ADHD is in the opposite direction to the effect on somatic insulinopathies. Our work might open up new directions for clinical and neuropsychopharmacological research by introducing insulin signalling as a possible mechanism underlying the multimorbidity of major mental disorders and somatic diseases. Further studies are warranted to investigate the biological meaning of the observed correlations and potential non-genetic effects contributing to insulin-related multimorbidity.

### URLs

LDSC, https://github.com/bulik/ldsc; Pre-computed European LD scores, https://data.broadinstitute.org/alkesgroup/LDSCORE/; GNOVA, https://github.com/xtonyjiang/GNOVA;

GWAS summary statistics - ADHD, AN, ASD, BD, OCD, MDD, TS: https://www.med.unc.edu/pgc/download-results/; AD: https://ctg.cncr.nl/software/summary_statistics; SCZ: http://walters.psycm.cf.ac.uk/; 2hGlu, FPG, FPI, HbA1c, HOMA-IR: https://www.magicinvestigators.org/downloads/; BMI: https://portals.broadinstitute.org/collaboration/giant/index.php/GIANT_consortium_data_files; MSigDB: https://www.gsea-msigdb.org/gsea/msigdb/index.jsp.

## Supplementary information

Supplementary information

Supplementary Tables

## Data Availability

Codes used for the analyses reported in this study are available under specific request to the first (Dr Giuseppe Fanelli) or corresponding author (Dr Janita Bralten).
