# Supplementary material for: Insulinopathies of the brain? Genetic overlap between somatic insulin-related and neuropsychiatric disorders
Source: Transl Psychiatry. 2022 Feb 14;12:59. doi: 10.1038/s41398-022-01817-0 (PMC8844407; doi:10.1038/s41398-022-01817-0)
Supplement: Supplementary file 1 — Supplementary information [file 41398_2022_1817_MOESM1_ESM.docx]

**Insulinopathies of the brain? Genetic overlap between somatic insulin-related and neuropsychiatric disorders**

Giuseppe Fanelli, M.D. ^1,2^, Barbara Franke, Ph.D. ^2,3^, Ward De Witte, B.Sc. ^2^, I. Hyun Ruisch, M.D. ^4^, Jan Haavik, M.D., Ph.D. ^5,6^, Veerle van Gils, M.Sc. ^7^, Willemijn J. Jansen, Ph.D. ^7^, Stephanie J. B. Vos, Ph.D. ^7^, Lars Lind, M.D., Ph.D. ^8^, Jan K. Buitelaar, M.D., Ph.D. ^9^, Tobias Banaschewski, M.D., Ph.D. ^10^, Søren Dalsgaard, M.D., Ph.D. ^11,12^, Alessandro Serretti, M.D., Ph.D. ^1^, Nina Roth Mota, Ph.D. ^2^, Geert Poelmans, M.D., Ph.D. ^2^, Janita Bralten, Ph.D. ^2^*

*^1^ Department of Biomedical and Neuromotor Sciences, University of Bologna, Bologna, Italy*

*^2^ Department of Human Genetics, Radboud University Medical Center, Donders Institute for Brain, Cognition and Behaviour, Nijmegen, The Netherlands*

*^3^ Department of Psychiatry, Radboud University Medical Center, Donders Institute for Brain, Cognition and Behaviour, Nijmegen, The Netherlands*

*^4^ Department of Child and Adolescent Psychiatry, University of Groningen, University Medical Center Groningen, Groningen, The Netherlands*

*^5^ Department of Biomedicine, University of Bergen, Bergen, Norway*

^6^ *Division of Psychiatry, Haukeland University Hospital, Bergen, Norway*

*^7^ Department of Psychiatry and Neuropsychology, School for Mental Health and Neuroscience, Maastricht University, Maastricht, The Netherlands*

*^8^ Department of Medical Sciences, Uppsala University, Uppsala, Sweden*

*^9^ Department of Cognitive Neuroscience, Donders Institute for Brain, Cognition and Behaviour, Radboud University Medical Center, Nijmegen, The Netherlands*

*^10^ Department of Child and Adolescent Psychiatry and Psychotherapy, Central Institute of Mental Health, Medical Faculty Mannheim, Heidelberg University, Mannheim, Germany*

*^11^ National Centre for Register-Based Research, Aarhus University, Aarhus, Denmark*

*^12^ The Lundbeck Foundation Initiative for Integrative Psychiatric Research, PSYCH, Aarhus, Denmark*

* *Corresponding author*:

Dr Janita Bralten

Radboud university medical center

Department of Human Genetics

P.O. Box 9101, 6500 HB Nijmegen

The Netherlands

E-mail: [janita.bralten@radboudumc.nl](mailto:janita.bralten@radboudumc.nl)

1. **Genome-wide bivariate genetic correlation estimations**

First, the GWAS summary statistics were quality-controlled through the Linkage Disequilibrium Score Regression (LDSC) *munge_sumstats.py* function by removing single nucleotide polymorphisms (SNPs) that had a MAF≤0.01 or were poorly imputed (INFO score≤0.9 or not matching to HapMap3 SNPs, which tended to be well imputed in most studies). Strand-ambiguous variants, missing or out-of-bounds values (expected ranges for MAF: 0-1, INFO: 0-1.5, p-value: 0-1), and all indels were also removed. If the sample size varied from one SNP to the others, SNPs having an effective sample size [Neff = 4/(1/Cases + 1/Controls)] less than 0.67 times the 90^th^ percentile of sample size were removed. Pre-computed Linkage Disequilibrium (LD) scores, referring to the European population from the 1,000 Genomes Project (1kGP), were used as regression weights to estimate genetic correlations in LDSC. The major histocompatibility complex (MHC) locus (chr6:29-33 Mbp) was excluded from LD scores computation because of its high regional LD and the risk of producing bias in the regression. Standard errors and statistical significance were assessed via a block jack-knife procedure.

1. **Genetic covariance analyses stratified by functional annotations**

GeNetic cOVariance Analyzer (GNOVA) uses the method of moments as underlying framework to partition genetic covariances by functionally annotated subsets of SNPs. Insulin signalling-related gene-sets were identified and downloaded from Molecular Database v7.1 (MSigDB) (<https://www.gsea-msigdb.org/gsea/msigdb/index.jsp>) by using “insulin” as search keyword. Eight curated (by BioCarta, Broad Institute, Kyoto Encyclopedia of Genes and Genomes (KEGG), Massachusetts Institute of Technology, MSigDB Team, US National Institutes of Health, Nature publishing group, and Reactome) gene-sets were selected that relate directly to insulin signalling and have insulin in their name headers (gene-set sizes ranged from 27 to 137 genes; see **Supplementary Table S1** for a complete list of genes included in each gene-set). We also considered an additional curated gene-set including 53 genes that are specifically relevant to central nervous system (CNS) insulin signalling, in addition to being listed among the top-ranked OCD GWAS findings or supported by animal/candidate gene/transcriptomic studies on OCD (**Supplementary Table S2**)^1^. To prevent circularity in our analyses, which might compromise statistical inferences and increase the likelihood of false positive findings, this gene-set related to CNS insulin-signalling was not used for stratified genetic covariance analyses for OCD. Each gene-set was annotated to SNP positions on the 1kGP reference panel using the LDSC *make_annot.py* function. Then we performed stratified genetic covariance analyses of AD, ASD, obsessive-compulsive disorder (OCD) with metabolic syndrome (MetS), obesity and/or type 2 diabetes mellitus (T2DM) in GNOVA, using the LDSC-munged GWAS summary statistics of the considered phenotypes as input datasets, as well as the annotated SNP positions, as indicated in <https://github.com/xtonyjiang/GNOVA>. In genetic covariance analyses, we expect that neither the number of genes in each gene-set nor the size of each gene in the gene-set will influence the chance of obtaining significant results, as an increase in the number of annotated SNPs for each gene-set may also increase the chance of finding effects of divergent localised genetic covariance, which ultimately results in a lower probability of finding an overall significant genetic covariance at the level of that gene-set. This is also evidenced by the fact that even in the absence of a genetic correlation at the genome-wide level (i.e., at the genome-wide level the analysis includes a much larger number of SNPs) between a pair of phenotypic traits, a genetic correlation at the local level (i.e., at the locus/gene-set level the analysis includes a lot fewer SNPs) may still be found, as evidenced by recent publications^2, 3^.

1. **Samples characteristics**

Below, we report a summary of the characteristics of each genome-wide association study (GWAS), whose summary results were used in our analyses. For additional details about the GWAS methodology, please refer to the original publication, whose reference is also provided here. For each GWAS dataset, **Table 1** lists the number of cases and controls and the N_eff_ (only for binary phenotypes).

- 1. ***Samples included in the analyses***

**Obesity**^4^

A population-based case-control sample of European ethnicity from UK Biobank, release 2, was used for this GWAS. Age, sex, Townsend deprivation index, batch, and the first 20 population principal components (PCs) were considered as covariates for the GWAS analysis. Three independent genome-wide loci associated with obesity were found. Cases were identified by referring to UK Biobank field 41204 (“Diagnoses - secondary ICD-10 (International Classification of Diseases, 10th revision”) and phenotype code “E66 Overweight and obesity”.

**Metabolic syndrome (MetS)**^5^

A population-based case-control sample of European ethnicity from UK Biobank was used for this GWAS, considering age, sex, batch, and the first population PCs as covariates. Ninety-three independent genome-wide loci associated with MetS were identified. The National Cholesterol Education Program Expert Panel (NCEP) criteria for MetS were adopted to identify the five components of the syndrome. Three out of the following five criteria had to be met: high-density lipoprotein (HDL) cholesterol <1.0 mmol/L in men and <1.3 mmol/L in women, serum triglycerides ≥1.7 mmol/L, serum glucose ≥6.1 mmol/L or undergoing antidiabetic treatment, blood pressure ≥130/85 mmHg or undergoing antihypertensive treatment, waist circumference >102 cm in men and >88 cm in women.

**Type 2 diabetes mellitus (T2DM)**^6^

GWAS results for 32 studies, including individuals of European descendent, were aggregated through this case-control meta-analysis. Within each cohort, all polymorphisms were tested for their association with T2DM without adjusting for BMI and in a sex-combined manner, with further study-specific covariates. Two hundred and forty-three risk loci associated with T2DM were identified. The T2DM diagnosis was obtained in accordance with the World Health Organisation (WHO) 1999 criteria or ICD-9 codes, based on hospital discharge diagnoses or electronic health records. The phenotype was defined based on a fasting glucose history ≥7.0 mmol/l or a 2-hour glucose ≥11.1 mmol/l during an oral glucose tolerance test (OGTT) or HbA1c ≥6.5% or use of antidiabetic drugs. Alternatively, in UK Biobank the phenotype was based on self-reported diabetes confirmed by an additional validated questionnaire. In order to prevent mixing with type 1 diabetes mellitus (T1DM), all patients were over 35 years of age at onset and had no detectable antibodies to glutamic acid decarboxylase (anti-GAD) or fasting C-peptide ≤0.30 nmol/L. Individuals were also excluded if they had known or probable first-degree familiarity for T1DM, or whether insulin treatment was started within ten years after the diagnosis.

**Alzheimer’s disease (AD)**^7^

This GWAS meta-analysis comprised 13 population-based, twin or case-control cohorts, including individuals of European ancestry, and identified 38 independent genomic risk loci associated with AD. GWASs on individual cohorts partly considered sex and age as covariates, along with the first population PCs. The diagnosis of AD followed the criteria for definite, probable, or possible AD of the National Institute of Neurological and Communicative Disorders and Stroke and the AD and Related Disorders Association (NINCDS-ADRDA), the criteria of the National Institute on Aging-Alzheimer's Association (NIA/AA), the ICD-10 codes G30 or F00, the ICD-9 code 331.0, or it was obtained from population-based health care registers including those with information on causes of death or prescribed drugs. With regard to the UK Biobank, raw data was also used to create a proxy weighted AD phenotype. In detail, in UK Biobank this phenotype was constructed as a linear count of the number of affected biological parents (0, 1 or 2). Each unaffected parent's contribution to this count was weighted by their age/age at death to account for the possibility that they have not yet passed through the risk period for this late-onset disease. Participants with a diagnosis of “Alzheimer's disease” (code G30) or “Dementia in Alzheimer's disease” (chapter on mental and behavioural disorders; code F00) as the cause of death or from previous hospitalisation records were treated as AD cases and obtained the highest possible risk score of 2, regardless of pathological family history. Due to the small number of the latter cases with a direct diagnosis in UK biobank (393 in all), this information was used to complement the proxy parent phenotype instead of as a primary outcome.

**Autism spectrum disorder (ASD)**^8^

Five family-based cohorts (i.e., the Geschwind Autism Center of Excellence (ACE), the Autism Genome Project (AGP), the Autism Genetic Resource Exchange (AGRE), the US National Institute of Mental Health (NIMH) Repository, the Montreal/Boston Collection (MONBOS), and the Simons Simplex Collection) of European ancestry as well as the Lundbeck Foundation Initiative for Integrative Psychiatric Research” - iPSYCH Danish population-based case-control sample were meta-analysed. The first PCs were included as covariates in the GWAS conducted in the iPSYCH sample, whereas the trios subsamples did not require PCs covariates. Five loci were genome-wide associated with ASD after the meta-analysis. With regard to the included family-based studies, the diagnosis was formulated using usual research tools and consensus diagnoses by clinicians. In the population-based cohort, cases were identified through the Danish Central Psychiatric Research Registry and diagnosed by a psychiatrist according to ICD-10 criteria for atypical autism (F84.1), childhood autism (F84.0), Asperger's syndrome (F84.5), pervasive developmental disorder, unspecified (F84.9), and other pervasive developmental disorders (F84.8).

**Obsessive-compulsive disorder (OCD)**^9^

In this meta-analysis, two case-control and parent-offspring trios cohorts (International Obsessive Compulsive Disorder Foundation Genetics Collaborative (IOCDF-GC), and OCD Collaborative Genetics Association Studies (OCGAS)), including individuals of European ancestry, were combined. No locus was found to be genome-wide associated with OCD. The diagnosis was obtained in compliance with the DSM-IV (Diagnostic and Statistical Manual of Mental Disorders, 4th edition) criteria.

**Body mass index (BMI)**^10^

Data from the population-based UK Biobank-release 2 cohort, which includes individuals of European origin, where used for this GWAS. Only the genotyping array was included as a covariate for the GWAS. Eight hundred and ninety-eight independent risk loci were identified. The phenotype considered was a continuous measure of BMI.

**Glucose levels 2 hours after an oral glucose challenge (2hGlu)**^11^

Summary data for 2hGlu are derived from a meta-analysis of nine GWAS studies and a follow-up of 29 independent loci in 17 other studies, all including non-diabetic individuals of European ethnicity. The GWAS analyses were adjusted for BMI, age, sex, and study-specific covariates (primarily PCs and/or recruitment centres). The 2hGlu measurements were untransformed and adjusted for age, sex, BMI, and study-specific covariates. Individuals with diabetes (identified by previous diagnosis, ongoing antidiabetic treatment or fasting plasma glucose ≥7 mmol/L) were excluded from the study.

**Fasting plasma glucose (FPG) and insulin (FPI)**^12^

GWAS meta-analyses on fasting glucose levels (FPG) measured in mmol/L and fasting plasma insulin concentrations (FPI) measured in pmol/L were conducted in individuals of European ancestry. Data for FPG and FPI were analysed without adjusting for BMI, but considering PCs, age, sex, and recruitment centres as covariates. Individuals with a medical diagnosis of diabetes, or treated with oral antidiabetic medications or insulin, or having a FPG ≥7 mmol/L were excluded from the analyses. Some individual studies applied additional sample exclusions, such as pregnancy status, non-fasting individuals, and diagnosis of T1DM.

**Glycated haemoglobin (HbA1c)**^13^

Fifty-six population-based, prospective, case-control, or twin GWAS studies, including individuals of European descent, were meta-analysed. Age, sex, and PCs were included as GWAS covariates in most of the individual samples. Forty-three independent loci were identified in European individuals. All participants were diabetes-free (as defined by physician diagnosis, medication use, or FPG ≥7 mmol/L). When FPG was not available, some single studies also eliminated subjects having 2hGlu ≥11.1 mmol/L, or HbA1c ≥6.5%.

**Homeostatic model assessment for insulin resistance (HOMA-IR)**^14^

Data from 20 GWAS, whose participants were all adults of European ancestry, were meta-analysed. In each cohort, regression models were adjusted for sex, age, and recruitment centres if applicable. One locus was associated with HOMA-IR. Individuals were excluded from the analyses if they were diagnosed with T2DM, were being treated with oral anti-diabetic drugs or insulin or had FPG ≥7 mmol/L. Individual studies applied additional sample exclusions, including pregnancy, non-fasting individuals, T1DM, or outliers ±3 standard deviations of the distribution for FPG or FPI.

**Attention-deficit/hyperactivity disorder (ADHD)**^15^

The GWAS summary data were referred to a European subsample from the meta-analysis on ADHD, which was conducted on samples from the Psychiatric Genomics Consortium (PGC) and iPSYCH. In each cohort, GWASs were corrected for population stratification by using relevant PCs as covariates, and batch effect (if applicable). The overall analysis, which considered 12 cohorts, revealed 12 loci with ADHD. For the iPSYCH cohort, cases were identified using the Danish Psychiatric Central Research Register and diagnoses were conﬁrmed by expert clinicians in accordance with ICD-10. The PGC cohorts included seven case-control and four family-based cohorts. Patients with ADHD were recruited from hospitals and clinics or by making use of medical registers. Diagnoses were made by trained personnel using research diagnostic tools.

**Anorexia nervosa (AN)**^16^

Thirty-three GWAS studies, including European individuals from the Anorexia Nervosa Genetics Initiative (ANGI), the Eating Disorders Working Group of the Psychiatric Genomics Consortium (PGC-ED) and UK Biobank cohorts as well as additional controls from Poland were combined in a meta-analysis on AN. The first five PCs and those significantly associated with the phenotype were included as covariates in the GWAS analyses. Eight significant loci were identified. The AN phenotype was defined by referring to medical records from hospitals and other registries, structured clinical interviews or online questionnaires based on standardised diagnostic criteria (from the DSM-III-R, DSM-IV, ICD-8, ICD-9, ICD-10), or self-reported diagnoses for UK Biobank participants.

**Bipolar disorder (BD)**^17^

Fifty-seven case-control studies from Australia, North America, and Europe, including individuals of European descent, were meta-analysed. Population PCs were used as covariates in the GWAS analyses. Sixty-four independent genomic loci were genome-wide associated with BD. A lifetime diagnosis of BD was formulated according to the criteria indicated by the DSM-IV, ICD-9, or ICD-10, through the use of structured clinical interviews, medically administered checklists, or a review of medical records.

**Major depressive disorder (MDD)**^18, 19^

Summary data were derived from a GWAS meta-analysis of European individuals from the 33 cohorts of the Psychiatric Genomics Consortium (excluding UK Biobank and 23andMe data) as described in Wray et al., 2018 ^18^ and the broad depression phenotype in the full release of the UK Biobank as described in Howard et al., 2018 ^19^. Age, sex, batch effects, population PCs were considered as covariates in the GWAS analyses. The overall meta-analysis identified one-hundred and one independent loci. With regard to the PGC cohorts, cases were identified using structured diagnostic interviews (referring to standardised clinical criteria from DSM-V, ICD-9, and ICD-10), or review of electronic health records. In UK Biobank, the broad depression phenotype was obtained using self-reported help-seeking behaviour for mental health difﬁculties and affirmative response at least to one of the following two questions: “Have you ever seen a general practitioner (GP) for nerves, anxiety, tension or depression?” (ﬁeld 2090) or “Have you ever seen a psychiatrist for nerves, anxiety, tension or depression?” (ﬁeld 2010); alternatively, primary, or secondary diagnoses of a depressive mood disorder from hospital medical records were used (ﬁelds 41202 and 41204; ICD codes: F32—Single Episode Depression, F33—Recurrent Depression, F34—Persistent mood disorders, F38—Other mood disorders and F39—Unspeciﬁed mood disorders).

**Schizophrenia (SCZ)**^20^

The summary data were referred to a meta-analysis of SCZ GWAS data from 46 case-control cohorts of European descendent. In this GWAS meta-analysis, PGC data were reanalysed by including a larger CLOZUK subsample. The first population PCs were considered as covariates in the GWAS analyses. The meta-analysis identified one-hundred and forty-five loci associated with SCZ. Cases included patients either with SCZ or schizoaffective disorder, and they were identified by clinical diagnosis or using research-based assessment tools, depending on the cohort.

**Tourette’s syndrome (TS)**^21^

The GWAS meta-analysis on TS included three case-control cohorts and one family-based cohort from Europe and North America, including individuals of European. The first multidimensional scaling components were included as covariates in the GWAS. One genome-wide significant locus was associated with TS. Most of the cases were diagnosed in accordance with DSM-IV-TR or DSM-V criteria for TS. Twelve cases met DSM-V criteria for chronic vocal or motor tic disorder. All cases were recruited in specialised clinics or by online recruitment combined with web-based, validated phenotypic assessments.

**References**

1. van de Vondervoort I, Poelmans G, Aschrafi A, Pauls DL, Buitelaar JK, Glennon JC *et al.* An integrated molecular landscape implicates the regulation of dendritic spine formation through insulin-related signalling in obsessive-compulsive disorder. *J Psychiatry Neurosci* 2016; **41**(4)**:** 280-285.

2. van Rheenen W, Peyrot WJ, Schork AJ, Lee SH, Wray NR. Genetic correlations of polygenic disease traits: from theory to practice. *Nat Rev Genet* 2019; **20**(10)**:** 567-581.

3. Werme J, van der Sluis S, Posthuma D, de Leeuw CA. LAVA: An integrated framework for local genetic correlation analysis. *bioRxiv* 2021**:** 2020.2012.2031.424652.

4. Watanabe K, Stringer S, Frei O, Umicevic Mirkov M, de Leeuw C, Polderman TJC *et al.* A global overview of pleiotropy and genetic architecture in complex traits. *Nat Genet* 2019; **51**(9)**:** 1339-1348.

5. Lind L. Genome-Wide Association Study of the Metabolic Syndrome in UK Biobank. *Metab Syndr Relat Disord* 2019; **17**(10)**:** 505-511.

6. Mahajan A, Taliun D, Thurner M, Robertson NR, Torres JM, Rayner NW *et al.* Fine-mapping type 2 diabetes loci to single-variant resolution using high-density imputation and islet-specific epigenome maps. *Nat Genet* 2018; **50**(11)**:** 1505-1513.

7. Wightman DP, Jansen IE, Savage JE, Shadrin AA, Bahrami S, Holland D *et al.* A genome-wide association study with 1,126,563 individuals identifies new risk loci for Alzheimer's disease. *Nat Genet* 2021; **53**(9)**:** 1276-1282.

8. Grove J, Ripke S, Als TD, Mattheisen M, Walters RK, Won H *et al.* Identification of common genetic risk variants for autism spectrum disorder. *Nat Genet* 2019; **51**(3)**:** 431-444.

9. International Obsessive Compulsive Disorder Foundation Genetics C, Studies OCDCGA. Revealing the complex genetic architecture of obsessive-compulsive disorder using meta-analysis. *Mol Psychiatry* 2018; **23**(5)**:** 1181-1188.

10. Pulit SL, Stoneman C, Morris AP, Wood AR, Glastonbury CA, Tyrrell J *et al.* Meta-analysis of genome-wide association studies for body fat distribution in 694 649 individuals of European ancestry. *Hum Mol Genet* 2019; **28**(1)**:** 166-174.

11. Saxena R, Hivert MF, Langenberg C, Tanaka T, Pankow JS, Vollenweider P *et al.* Genetic variation in GIPR influences the glucose and insulin responses to an oral glucose challenge. *Nat Genet* 2010; **42**(2)**:** 142-148.

12. Lagou V, Magi R, Hottenga JJ, Grallert H, Perry JRB, Bouatia-Naji N *et al.* Sex-dimorphic genetic effects and novel loci for fasting glucose and insulin variability. *Nat Commun* 2021; **12**(1)**:** 24.

13. Wheeler E, Leong A, Liu CT, Hivert MF, Strawbridge RJ, Podmore C *et al.* Impact of common genetic determinants of Hemoglobin A1c on type 2 diabetes risk and diagnosis in ancestrally diverse populations: A transethnic genome-wide meta-analysis. *PLoS Med* 2017; **14**(9)**:** e1002383.

14. Dupuis J, Langenberg C, Prokopenko I, Saxena R, Soranzo N, Jackson AU *et al.* New genetic loci implicated in fasting glucose homeostasis and their impact on type 2 diabetes risk. *Nat Genet* 2010; **42**(2)**:** 105-116.

15. Demontis D, Walters RK, Martin J, Mattheisen M, Als TD, Agerbo E *et al.* Discovery of the first genome-wide significant risk loci for attention deficit/hyperactivity disorder. *Nat Genet* 2019; **51**(1)**:** 63-75.

16. Watson HJ, Yilmaz Z, Thornton LM, Hubel C, Coleman JRI, Gaspar HA *et al.* Genome-wide association study identifies eight risk loci and implicates metabo-psychiatric origins for anorexia nervosa. *Nat Genet* 2019; **51**(8)**:** 1207-1214.

17. Mullins N, Forstner AJ, O'Connell KS, Coombes B, Coleman JRI, Qiao Z *et al.* Genome-wide association study of more than 40,000 bipolar disorder cases provides new insights into the underlying biology. *Nat Genet* 2021; **53**(6)**:** 817-829.

18. Wray NR, Ripke S, Mattheisen M, Trzaskowski M, Byrne EM, Abdellaoui A *et al.* Genome-wide association analyses identify 44 risk variants and refine the genetic architecture of major depression. *Nat Genet* 2018; **50**(5)**:** 668-681.

19. Howard DM, Adams MJ, Shirali M, Clarke TK, Marioni RE, Davies G *et al.* Genome-wide association study of depression phenotypes in UK Biobank identifies variants in excitatory synaptic pathways. *Nat Commun* 2018; **9**(1)**:** 1470.

20. Pardinas AF, Holmans P, Pocklington AJ, Escott-Price V, Ripke S, Carrera N *et al.* Common schizophrenia alleles are enriched in mutation-intolerant genes and in regions under strong background selection. *Nat Genet* 2018; **50**(3)**:** 381-389.

21. Yu D, Sul JH, Tsetsos F, Nawaz MS, Huang AY, Zelaya I *et al.* Interrogating the Genetic Determinants of Tourette's Syndrome and Other Tic Disorders Through Genome-Wide Association Studies. *Am J Psychiatry* 2019; **176**(3)**:** 217-227.
