# Supplementary material for: Insulinopathies of the brain? Genetic overlap between somatic insulin-related and neuropsychiatric disorders
Source: Transl Psychiatry. 2022 Feb 14;12:59. doi: 10.1038/s41398-022-01817-0 (PMC8844407; doi:10.1038/s41398-022-01817-0)
Supplement: Supplementary file 2 — Supplementary Tables [file 41398_2022_1817_MOESM2_ESM.docx]

[Table S1. List of genes included in each gene-set downloaded from Molecular signature Database (MSigDB). 3](#_Toc92884346)

[Table S2. List of genes included in van de Vondervoort's gene-set according to the 2020 update, as provided by the authors. 4](#_Toc92884347)

[Table S3 (a). Results of the genetic covariance analyses between Alzheimer's disease and metabolic syndrome stratified by insulin signalling gene-sets. 4](#_Toc92884348)

[Table S3 (b). Results of the genetic covariance analyses between Alzheimer's disease and obesity stratified by insulin signalling gene-sets. 6](#_Toc92884349)

[Table S3 (c). Results of the genetic covariance analyses between Alzheimer's disease and type 2 diabetes mellitus stratified by insulin signalling gene-sets. 7](#_Toc92884350)

[Table S4 (a). Results of the genetic covariance analyses between anorexia nervosa and metabolic syndrome stratified by insulin signalling gene-sets. 8](#_Toc92884351)

[Table S4 (b). Results of the genetic covariance analyses between anorexia nervosa and obesity stratified by insulin signalling gene-sets. 9](#_Toc92884352)

[Table S4 (c). Results of the genetic covariance analyses between anorexia nervosa and type 2 diabetes mellitus stratified by insulin signalling gene-sets. 10](#_Toc92884353)

[Table S5 (a). Results of the genetic covariance analyses between attention-deficit/hyperactivity disorder and metabolic syndrome stratified by insulin signalling gene-sets. 11](#_Toc92884354)

[Table S5 (b). Results of the genetic covariance analyses between attention-deficit/hyperactivity disorder and obesity stratified by insulin signalling gene-sets. 12](#_Toc92884355)

[Table S5 (c). Results of the genetic covariance analyses between attention-deficit/hyperactivity disorder and type 2 diabetes mellitus stratified by insulin signalling gene-sets. 13](#_Toc92884356)

[Table S6 (a). Results of the genetic covariance analyses between autism spectrum disorder and metabolic syndrome stratified by insulin signalling gene-sets. 14](#_Toc92884357)

[Table S6 (b). Results of the genetic covariance analyses between autism spectrum disorder and obesity stratified by insulin signalling gene-sets. 15](#_Toc92884358)

[Table S6 (c). Results of the genetic covariance analyses between autism spectrum disorder and type 2 diabetes mellitus stratified by insulin signalling gene-sets. 16](#_Toc92884359)

[Table S7 (a). Results of the genetic covariance analyses between bipolar disorder and metabolic syndrome stratified by insulin signalling gene-sets. 17](#_Toc92884360)

[Table S7 (b). Results of the genetic covariance analyses between bipolar disorder and obesity stratified by insulin signalling gene-sets. 18](#_Toc92884361)

[Table S7 (c). Results of the genetic covariance analyses between bipolar disorder and type 2 diabetes mellitus stratified by insulin signalling gene-sets. 19](#_Toc92884362)

[Table S8 (a). Results of the genetic covariance analyses between major depressive disorder and metabolic syndrome stratified by insulin signalling gene-sets. 20](#_Toc92884363)

[Table S8 (b). Results of the genetic covariance analyses between major depressive disorder and obesity stratified by insulin signalling gene-sets. 21](#_Toc92884364)

[Table S8 (c). Results of the genetic covariance analyses between major depressive disorder and type 2 diabetes mellitus stratified by insulin signalling gene-sets. 22](#_Toc92884365)

[Table S9 (a). Results of the genetic covariance analyses between obsessive-compulsive disorder and metabolic syndrome stratified by insulin signalling gene-sets. 23](#_Toc92884366)

[Table S9 (b). Results of the genetic covariance analyses between obsessive-compulsive disorder and obesity stratified by insulin signalling gene-sets. 24](#_Toc92884367)

[Table S9 (c). Results of the genetic covariance analyses between obsessive-compulsive disorder and type 2 diabetes mellitus stratified by insulin signalling gene-sets. 25](#_Toc92884368)

[Table S10 (a). Results of the genetic covariance analyses between Tourette’s syndrome and metabolic syndrome stratified by insulin signalling gene-sets. 26](#_Toc92884369)

[Table S10 (b). Results of the genetic covariance analyses between Tourette’s syndrome and obesity stratified by insulin signalling gene-sets. 27](#_Toc92884370)

[Table S10 (c). Results of the genetic covariance analyses between Tourette’s syndrome and type 2 diabetes mellitus stratified by insulin signalling gene-sets. 28](#_Toc92884371)

[Table S11 (a). Results of the genetic covariance analyses between schizophrenia and metabolic syndrome stratified by insulin signalling gene-sets. 29](#_Toc92884372)

[Table S11 (b). Results of the genetic covariance analyses between schizophrenia and obesity stratified by insulin signalling gene-sets. 30](#_Toc92884373)

[Table S11 (c). Results of the genetic covariance analyses between schizophrenia and type 2 diabetes mellitus stratified by insulin signalling gene-sets. 31](#_Toc92884374)

# **Table S1.** List of genes included in each gene-set downloaded from Molecular signature Database (MSigDB).

| REACTOME INSULIN PROCESSING | *CLTRN, CPE, ERO1A, ERO1B, EXOC1, EXOC2, EXOC3, EXOC4, EXOC5, EXOC6, EXOC7, EXOC8, INS, KIF5A, KIF5B, KIF5C, MYO5A, MYRIP, PCSK1, PCSK2, RAB27A, SLC30A5, SLC30A6, SLC30A7, SLC30A8, STX1A, VAMP2,* (synonymous genes: *NX-17, NX17, TMEM27, ERO1A, ERO1-L, ERO1-L-alpha, ERO1-alpha, ERO1L, ERO1LA, Ero1alpha, ERO1B, ERO1LB, Ero1beta*) |
| --- | --- |
| KEGG INSULIN SIGNALING PATHWAY | *ACACA, ACACB, AKT1, AKT2, AKT3, ARAF, BAD, BRAF, CALM1, CALM2, CALM3, CALML3, CALML5, CALML6, CBL, CBLB, CBLC, CRK, CRKL, EIF4E, EIF4E1B, EIF4E2, EIF4EBP1, ELK1, EXOC7, FASN, FBP1, FBP2, FLOT1, FLOT2, FOXO1, G6PC, G6PC2, GCK, GRB2, GSK3B, GYS1, GYS2, HK1, HK2, HK3, HRAS, IKBKB, INPP5D, INPP5K, INS, INSR, IRS1, IRS2, IRS4, KRAS, LIPE, MAP2K1, MAP2K2, MAPK1, MAPK10, MAPK3, MAPK8, MAPK9, MKNK1, MKNK2, MTOR, NRAS, PCK1, PCK2, PDE3A, PDE3B, PDPK1, PHKA1, PHKA2, PHKB, PHKG1, PHKG2, PIK3CA, PIK3CB, PIK3CD, PIK3CG, PIK3R1, PIK3R2, PIK3R3, PIK3R5, PKLR, PPARGC1A, PPP1CA, PPP1CB, PPP1CC, PPP1R3A, PPP1R3B, PPP1R3C, PPP1R3D, PRKAA1, PRKAA2, PRKAB1, PRKAB2, PRKACA, PRKACB, PRKACG, PRKAG1, PRKAG2, PRKAG3, PRKAR1A, PRKAR1B, PRKAR2A, PRKAR2B, PRKCI, PRKCZ, PRKX, PTPN1, PTPRF, PYGB, PYGL, PYGM, RAF1, RAPGEF1, RHEB, RHOQ, RPS6, RPS6KB1, RPS6KB2, RPTOR, SH2B2, SHC1, SHC2, SHC3, SHC4, SLC2A4, SOCS1, SOCS2, SOCS3, SOCS4, SORBS1, SOS1, SOS2, SREBF1, TRIP10, TSC1, TSC2* |
| BIOCARTA INSULIN PATHWAY | *CSNK2A1, ELK1, FOS, GRB2, HRAS, INSR, IRS1, JUN, MAP2K1, MAPK3, MAPK8, PIK3CA, PIK3CG, PIK3R1, PTPN11, RAF1, RASA1, SHC1, SLC2A4, SOS1, SRF* |
| PID INSULIN PATHWAY | *AKT1, AKT2, CAV1, CBL, CRK, DOK1, EIF4EBP1, EXOC1, EXOC2, EXOC3, EXOC4, EXOC5, EXOC6, EXOC7, F2RL2, FOXO3, GRB10, GRB14, GRB2, HRAS, INS, INSR, IRS1, NCK1, NCK2, PARD6A, PDPK1, PIK3CA, PIK3R1, PRKCI, PRKCZ, PTPN1, PTPN11, PTPRA, RAPGEF1, RASA1, RHOQ, RPS6KB1, SGK1, SH2B2, SHC1, SORBS1, SOS1, TRIP10* |
| REACTOME SIGNALING BY INSULIN RECEPTOR | *AKT2, ATP6AP1, ATP6V0A1, ATP6V0A2, ATP6V0A4, ATP6V0B, ATP6V0C, ATP6V0D1, ATP6V0D2, ATP6V0E1, ATP6V0E2, ATP6V1A, ATP6V1B1, ATP6V1B2, ATP6V1C1, ATP6V1C2, ATP6V1D, ATP6V1E1, ATP6V1E2, ATP6V1F, ATP6V1G1, ATP6V1G2, ATP6V1G3, ATP6V1H, FGF1, FGF10, FGF16, FGF17, FGF18, FGF19, FGF2, FGF20, FGF22, FGF23, FGF3, FGF4, FGF5, FGF6, FGF7, FGF8, FGF9, FGFR1, FGFR2, FGFR3, FGFR4, FLT3, FLT3LG, FRS2, GAB1, GAB2, GRB10, GRB2, HRAS, INS, INSR, IRS1, IRS2, KL, KLB, KRAS, MAPK1, MAPK3, NRAS, PDE3B, PDPK1, PIK3C3, PIK3CA, PIK3CB, PIK3R1, PIK3R2, PIK3R4, PTPN11, SHC1, SOS1, TCIRG1, THEM4, TLR9, TRIB3* |
| REACTOME INSULIN RECEPTOR SIGNALLING CASCADE | *AKT2, FGF1, FGF10, FGF16, FGF17, FGF18, FGF19, FGF2, FGF20, FGF22, FGF23, FGF3, FGF4, FGF5, FGF6, FGF7, FGF8, FGF9, FGFR1, FGFR2, FGFR3, FGFR4, FLT3, FLT3LG, FRS2, GAB1, GAB2, GRB10, GRB2, HRAS, INS, INSR, IRS1, IRS2, KL, KLB, KRAS, MAPK1, MAPK3, NRAS, PDE3B, PDPK1, PIK3C3, PIK3CA, PIK3CB, PIK3R1, PIK3R2, PIK3R4, PTPN11, SHC1, SOS1, THEM4, TLR9, TRIB3* |
| REACTOME REGULATION OF INSULIN SECRETION | *ABCC8, AC093503.1, ACSL3, ACSL4, ADCY5, ADCY6, ADCY8, ADRA2A, ADRA2C, AHCYL1, AKAP5, CACNA1A, CACNA1C, CACNA1D, CACNA1E, CACNA2D2, CACNB2, CACNB3, CD36, CHRM3, FFAR1, GCG, GLP1R, GNA11, GNA14, GNA15, GNAI1, GNAI2, GNAQ, GNAS, GNB1, GNB2, GNB3, GNB4, GNB5, GNG10, GNG11, GNG12, GNG13, GNG2, GNG3, GNG4, GNG5, GNG7, GNGT1, GNGT2, INS, IQGAP1, ITPR1, ITPR2, ITPR3, KCNB1, KCNC2, KCNG2, KCNJ11, KCNS3, MARCKS, PLCB1, PLCB2, PLCB3, PRKACA, PRKACB, PRKACG, PRKAR1A, PRKAR1B, PRKAR2A, PRKAR2B, PRKCA, RAP1A, RAPGEF3, RAPGEF4, SLC2A1, SLC2A2, SNAP25, STX1A, STXBP1, SYT5, VAMP2* |
| REACTOME INSULIN RECEPTOR RECYCLING | *ATP6AP1, ATP6V0A1, ATP6V0A2, ATP6V0A4, ATP6V0B, ATP6V0C, ATP6V0D1, ATP6V0D2, ATP6V0E1, ATP6V0E2, ATP6V1A, ATP6V1B1, ATP6V1B2, ATP6V1C1, ATP6V1C2, ATP6V1D, ATP6V1E1, ATP6V1E2, ATP6V1F, ATP6V1G1, ATP6V1G2, ATP6V1G3, ATP6V1H, INS, INSR, TCIRG1* |

# **Table S2.** List of genes included in van de Vondervoort's gene-set according to the 2020 update, as provided by the authors.

Abbreviations: OCD, obsessive-compulsive disorder; GWAS, genome-wide association study.

| OCD GWAS genes | *ADD3, ARHGAP15, BTBD3, CCNC, CYTIP, DCC, DLGAP1, DNAI1, DOCK1, EBF2, EFNA5, EREG, GJD2, GNRH1, IGF1R, IRS2, ITGA9, KCNB2, KCNQ1, LNX1, MEIS2, MTUS2, NSG2* (synonymous: *HMP19*)*, PDE4D, PRDM13, RACGAP1, REXO1, SEMA4D, SERPINH1, SLIT3, SORBS1, TBP, TFDP2, TMEM252, TRIOBP, TSPAN14, TXNL1, UBL3, ZBTB43, ZFP64* |
| --- | --- |
| other OCD candidate genes | *BDNF, DLGAP3, FKBP1A, GRIN2B, HOXB8, HTR1B, HTR2C, IGF1, INSR, NOS1, SLC1A1, SLITRK5, TNF* (synonymous: *TNFA*) |

** *HTR2C* is on the Xq23 locus, and it was excluded from the analyses.

* *IGF1* and *INSR* were later added to the molecular landscape by the authors who curated the gene set (update 2020)

# **Table S3 (a).** Results of the genetic covariance analyses between Alzheimer's disease and metabolic syndrome stratified by insulin signalling gene-sets.

* Nominally significant stratified genetic covariance estimates (P < 0.05)

** Statistically significant stratified genetic covariance estimates (P < 2.06x10-4)

| *Gene-set name* | *n genes/gene-set* | *ρ_g_ corrected* | *SE ρ_g_* | *P corrected* | *r_g_ corrected* | *h^2^_SNP_ 1* | *h^2^_SNP_ 2* | *annotated SNPs* | *total SNPs* |
| --- | --- | --- | --- | --- | --- | --- | --- | --- | --- |
| BIOCARTA INSULIN PATHWAY | 21 | -0.00018 | 0.00007 | 0.00608 * | -0.72549 | 0.00008 | 0.00076 | 1,415 | 942,214 |
| KEGG INSULIN SIGNALING PATHWAY | 137 | 0.00030 | 0.00026 | 0.25606 | 0.08886 | 0.00496 | 0.00223 | 11,202 | 942,214 |
| PID INSULIN PATHWAY | 44 | 0.00020 | 0.00010 | 0.05917 | 0.47389 | 0.00018 | 0.00092 | 3,987 | 942,214 |
| REACTOME INSULIN PROCESSING | 27 | 0.00005 | 0.00004 | 0.22737 | 0.27970 | 0.00006 | 0.00061 | 2,701 | 942,214 |
| REACTOME INSULIN RECEPTOR RECYCLING | 26 | 0.00019 | 0.00012 | 0.11322 | 0.71075 | 0.00009 | 0.00077 | 2,138 | 942,214 |
| REACTOME INSULIN RECEPTOR SIGNALLING CASCADE | 54 | 0.00035 | 0.00026 | 0.18057 | § NA | -0.00056 | 0.00100 | 4,426 | 942,214 |
| REACTOME REGULATION OF INSULIN SECRETION | 77 | 0.00143 | 0.00042 | 0.00060 * | 0.95033 | 0.00130 | 0.00173 | 8,927 | 942,214 |
| REACTOME SIGNALING BY INSULIN RECEPTOR | 78 | 0.00054 | 0.00035 | 0.12391 | § NA | -0.00043 | 0.00160 | 6,368 | 942,214 |
| van de Vondervoort's gene-set | 53 | 0.00055 | 0.00029 | 0.05853 | § NA | -0.00053 | 0.00076 | 8,047 | 942,214 |

*Abbreviations*

ρ_g_: genetic covariance estimate

ρ_g_ corrected: genetic covariance estimate with sample overlap correction #

SE ρ_g_: standard error of the estimate of ρ_g_

P: p-value from the statistical test for genetic covariance

P corrected: p-value from the statistical test for genetic covariance with sample overlap correction #

r_g_: genetic correlation estimate

r_g_ corrected: genetic correlation estimate with sample overlap correction #

h^2^_SNP_ 1: single-nucleotide polymorphism (SNP)-based heritability estimate for the first phenotype

h^2^_SNP_ 2: single-nucleotide polymorphism (SNP)-based heritability estimate for the second phenotype

# Alzheimer’s disease sample overlapping for the UK Biobank cohort with the obesity, metabolic syndrome and type 2 diabetes mellitus sample

§ Note: the heritability values derived from annotation stratified analyses may be sometimes negative, or very small. The noise in the estimation of single-nucleotide polymorphism (SNP)-based heritability may cause the genetic correlation estimates to be out of bounds (>1) or even be set as missing (NA) when the heritability estimates are small or below zero, respectively. In such cases, genetic covariance estimates are less biased than genetic correlation measures.

# **Table S3 (b).** Results of the genetic covariance analyses between Alzheimer's disease and obesity stratified by insulin signalling gene-sets.

* Nominally significant stratified genetic covariance estimates (P < 0.05)

** Statistically significant stratified genetic covariance estimates (P < 2.06x10-4)

| *Gene-set name* | *n genes/gene-set* | *ρ_g_ corrected* | *SE ρ_g_* | *P corrected* | *r_g_ corrected* | *h^2^_SNP_ 1* | *h^2^_SNP_ 2* | *annotated SNPs* | *total SNPs* |
| --- | --- | --- | --- | --- | --- | --- | --- | --- | --- |
| BIOCARTA INSULIN PATHWAY | 21 | -0.00029 | 0.00010 | 0.00282 * | § -1.36087 | 0.00008 | 0.00054 | 1,415 | 942,664 |
| KEGG INSULIN SIGNALING PATHWAY | 137 | -0.00076 | 0.00038 | 0.04538 * | -0.23454 | 0.00496 | 0.00214 | 11,202 | 942,664 |
| PID INSULIN PATHWAY | 44 | -0.00015 | 0.00013 | 0.24986 | -0.35878 | 0.00018 | 0.00101 | 3,987 | 942,664 |
| REACTOME INSULIN PROCESSING | 27 | -0.00008 | 0.00007 | 0.26759 | § NA | 0.00006 | -0.00044 | 2,701 | 942,664 |
| REACTOME INSULIN RECEPTOR RECYCLING | **26** | **-0.00079** | **0.00019** | **4.61x10-5 **** | **§ NA** | **0.00009** | **-0.00033** | **2,138** | **942,664** |
| REACTOME INSULIN RECEPTOR SIGNALLING CASCADE | 54 | -0.00009 | 0.00042 | 0.82578 | § NA | -0.00056 | 0.00307 | 4,426 | 942,664 |
| REACTOME REGULATION OF INSULIN SECRETION | 77 | 0.00120 | 0.00065 | 0.06477 | § 1.12271 | 0.00130 | 0.00089 | 8,927 | 942,664 |
| REACTOME SIGNALING BY INSULIN RECEPTOR | 78 | -0.00081 | 0.00055 | 0.14242 | § NA | -0.00043 | 0.00291 | 6,368 | 942,664 |
| van de Vondervoort's gene-set | 53 | 0.00049 | 0.00044 | 0.26340 | § NA | -0.00053 | 0.00016 | 8,047 | 942,664 |

*Abbreviations*

ρ_g_: genetic covariance estimate

ρ_g_ corrected: genetic covariance estimate with sample overlap correction #

SE ρ_g_: standard error of the estimate of ρ_g_

P: p-value from the statistical test for genetic covariance

P corrected: p-value from the statistical test for genetic covariance with sample overlap correction #

r_g_: genetic correlation estimate

r_g_ corrected: genetic correlation estimate with sample overlap correction #

h^2^_SNP_ 1: single-nucleotide polymorphism (SNP)-based heritability estimate for the first phenotype

h^2^_SNP_ 2: single-nucleotide polymorphism (SNP)-based heritability estimate for the second phenotype

# Alzheimer’s disease sample overlapping for the UK Biobank cohort with the obesity, metabolic syndrome and type 2 diabetes mellitus sample

§ Note: the heritability values derived from annotation stratified analyses may be sometimes negative, or very small. The noise in the estimation of single-nucleotide polymorphism (SNP)-based heritability may cause the genetic correlation estimates to be out of bounds (>1) or even be set as missing (NA) when the heritability estimates are small or below zero, respectively. In such cases, genetic covariance estimates are less biased than genetic correlation measures.

# **Table S3 (c).** Results of the genetic covariance analyses between Alzheimer's disease and type 2 diabetes mellitus stratified by insulin signalling gene-sets.

* Nominally significant stratified genetic covariance estimates (P < 0.05)

** Statistically significant stratified genetic covariance estimates (P < 2.06x10-4)

| *Gene-set name* | *n genes/gene-set* | *ρ_g_ corrected* | *SE ρ_g_* | *P corrected* | *r_g_ corrected* | *h^2^_SNP_ 1* | *h^2^_SNP_ 2* | *annotated SNPs* | *total SNPs* |
| --- | --- | --- | --- | --- | --- | --- | --- | --- | --- |
| BIOCARTA INSULIN PATHWAY | 21 | -0.00019 | 0.00007 | 0.00860 * | -0.95753 | 0.00008 | 0.00046 | 1,413 | 941,819 |
| KEGG INSULIN SIGNALING PATHWAY | 137 | -0.00066 | 0.00035 | 0.06160 | -0.19346 | 0.00489 | 0.00241 | 11,193 | 941,819 |
| PID INSULIN PATHWAY | 44 | 0.00010 | 0.00007 | 0.15122 | 0.22304 | 0.00018 | 0.00113 | 3,983 | 941,819 |
| REACTOME INSULIN PROCESSING | 27 | 0.00010 | 0.00004 | 0.00852 * | 0.30411 | 0.00006 | 0.00159 | 2,701 | 941,819 |
| REACTOME INSULIN RECEPTOR RECYCLING | 26 | 0.00027 | 0.00015 | 0.06374 | 0.75605 | 0.00010 | 0.00131 | 2,135 | 941,819 |
| REACTOME INSULIN RECEPTOR SIGNALLING CASCADE | 54 | 0.00009 | 0.00028 | 0.75908 | § NA | -0.00055 | 0.00216 | 4,421 | 941,819 |
| REACTOME REGULATION OF INSULIN SECRETION | 77 | 0.00140 | 0.00041 | 0.00064 * | 0.81279 | 0.00131 | 0.00227 | 8,920 | 941,819 |
| REACTOME SIGNALING BY INSULIN RECEPTOR | 78 | 0.00027 | 0.00041 | 0.50507 | § NA | -0.00042 | 0.00281 | 6,361 | 941,819 |
| van de Vondervoort's gene-set | 53 | -0.00002 | 0.00030 | 0.93867 | § NA | -0.00052 | 0.00265 | 8,035 | 941,819 |

*Abbreviations*

ρ_g_: genetic covariance estimate

ρ_g_ corrected: genetic covariance estimate with sample overlap correction #

SE ρ_g_: standard error of the estimate of ρ_g_

P: p-value from the statistical test for genetic covariance

P corrected: p-value from the statistical test for genetic covariance with sample overlap correction #

r_g_: genetic correlation estimate

r_g_ corrected: genetic correlation estimate with sample overlap correction #

h^2^_SNP_ 1: single-nucleotide polymorphism (SNP)-based heritability estimate for the first phenotype

h^2^_SNP_ 2: single-nucleotide polymorphism (SNP)-based heritability estimate for the second phenotype

# Alzheimer’s disease sample overlapping for the UK Biobank cohort with the obesity, metabolic syndrome and type 2 diabetes mellitus sample

§ Note: the heritability values derived from annotation stratified analyses may be sometimes negative, or very small. The noise in the estimation of single-nucleotide polymorphism (SNP)-based heritability may cause the genetic correlation estimates to be out of bounds (>1) or even be set as missing (NA) when the heritability estimates are small or below zero, respectively. In such cases, genetic covariance estimates are less biased than genetic correlation measures.

# **Table S4 (a).** Results of the genetic covariance analyses between anorexia nervosa and metabolic syndrome stratified by insulin signalling gene-sets.

* Nominally significant stratified genetic covariance estimates (P < 0.05)

** Statistically significant stratified genetic covariance estimates (P < 2.06x10-4)

| *Gene-set name* | *n genes/gene-set* | *ρ_g_ corrected* | *SE ρ_g_* | *P corrected* | *r_g_ corrected* | *h^2^_SNP_ 1* | *h^2^_SNP_ 2* | *annotated SNPs* | *total SNPs* |
| --- | --- | --- | --- | --- | --- | --- | --- | --- | --- |
| BIOCARTA INSULIN PATHWAY | 21 | -0.00009 | 0.00012 | 0.44676 | -0.12687 | 0.00078 | 0.00067 | 1,269 | 860,888 |
| KEGG INSULIN SIGNALING PATHWAY | 137 | -0.00134 | 0.00037 | 0.00026 * | -0.66128 | 0.00198 | 0.00207 | 9,968 | 860,888 |
| PID INSULIN PATHWAY | 44 | -0.00009 | 0.00023 | 0.69850 | -0.07049 | 0.00163 | 0.00101 | 3,897 | 860,888 |
| REACTOME INSULIN PROCESSING | 27 | 0.00015 | 0.00019 | 0.43304 | 0.10996 | 0.00215 | 0.00088 | 2,743 | 860,888 |
| REACTOME INSULIN RECEPTOR RECYCLING | 26 | -0.00013 | 0.00010 | 0.21307 | § NA | -0.00022 | 0.00065 | 1,825 | 860,888 |
| REACTOME INSULIN RECEPTOR SIGNALLING CASCADE | 54 | 0.00017 | 0.00021 | 0.42276 | 0.59523 | 0.00009 | 0.00086 | 3,755 | 860,888 |
| REACTOME REGULATION OF INSULIN SECRETION | 77 | -0.00085 | 0.00049 | 0.08283 | -0.38141 | 0.00350 | 0.00142 | 8,633 | 860,888 |
| REACTOME SIGNALING BY INSULIN RECEPTOR | 78 | 0.00004 | 0.00027 | 0.87434 | 0.09942 | 0.00013 | 0.00134 | 5,434 | 860,888 |
| van de Vondervoort's gene-set | 53 | -0.00108 | 0.00036 | 0.00281 * | -0.71072 | 0.00291 | 0.00079 | 8,102 | 860,888 |

*Abbreviations*

ρ_g_: genetic covariance estimate

ρ_g_ corrected: genetic covariance estimate with sample overlap correction #

SE ρ_g_: standard error of the estimate of ρ_g_

P: p-value from the statistical test for genetic covariance

P corrected: p-value from the statistical test for genetic covariance with sample overlap correction #

r_g_: genetic correlation estimate

r_g_ corrected: genetic correlation estimate with sample overlap correction #

h^2^_SNP_ 1: single-nucleotide polymorphism (SNP)-based heritability estimate for the first phenotype

h^2^_SNP_ 2: single-nucleotide polymorphism (SNP)-based heritability estimate for the second phenotype

# Anorexia nervosa sample overlapping for the UK Biobank cohort with the obesity, metabolic syndrome and type 2 diabetes mellitus sample

§ Note: the heritability values derived from annotation stratified analyses may be sometimes negative, or very small. The noise in the estimation of single-nucleotide polymorphism (SNP)-based heritability may cause the genetic correlation estimates to be out of bounds (>1) or even be set as missing (NA) when the heritability estimates are small or below zero, respectively. In such cases, genetic covariance estimates are less biased than genetic correlation measures.

# **Table S4 (b).** Results of the genetic covariance analyses between anorexia nervosa and obesity stratified by insulin signalling gene-sets.

* Nominally significant stratified genetic covariance estimates (P < 0.05)

** Statistically significant stratified genetic covariance estimates (P < 2.06x10-4)

| *Gene-set name* | *n genes/gene-set* | *ρ_g_ corrected* | *SE ρ_g_* | *P corrected* | *r_g_ corrected* | *h^2^_SNP_ 1* | *h^2^_SNP_ 2* | *annotated SNPs* | *total SNPs* |
| --- | --- | --- | --- | --- | --- | --- | --- | --- | --- |
| BIOCARTA INSULIN PATHWAY | 21 | 0.00011 | 0.00013 | 0.39006 | 0.19347 | 0.00079 | 0.00042 | 1,250 | 846,491 |
| KEGG INSULIN SIGNALING PATHWAY | 137 | -0.00044 | 0.00067 | 0.50986 | -0.23671 | 0.00199 | 0.00175 | 9,781 | 846,491 |
| PID INSULIN PATHWAY | 44 | -0.00035 | 0.00029 | 0.23318 | -0.29358 | 0.00157 | 0.00090 | 3,820 | 846,491 |
| REACTOME INSULIN PROCESSING | 27 | 0.00043 | 0.00022 | 0.05534 | § NA | 0.00214 | -0.00049 | 2,719 | 846,491 |
| REACTOME INSULIN RECEPTOR RECYCLING | 26 | -0.00039 | 0.00015 | 0.01118 * | § -1.77827 | -0.00019 | -0.00025 | 1,793 | 846,491 |
| REACTOME INSULIN RECEPTOR SIGNALLING CASCADE | 54 | -0.00032 | 0.00032 | 0.31039 | -0.49458 | 0.00014 | 0.00306 | 3,684 | 846,491 |
| REACTOME REGULATION OF INSULIN SECRETION | 77 | -0.00215 | 0.00079 | 0.00636 * | § -1.17431 | 0.00332 | 0.00101 | 8,450 | 846,491 |
| REACTOME SIGNALING BY INSULIN RECEPTOR | 78 | -0.00069 | 0.00042 | 0.09812 | -0.89784 | 0.00020 | 0.00294 | 5,335 | 846,491 |
| van de Vondervoort's gene-set | 53 | -0.00210 | 0.00063 | 0.00081 * | § NA | 0.00281 | -0.00008 | 7,834 | 846,491 |

*Abbreviations*

ρ_g_: genetic covariance estimate

ρ_g_ corrected: genetic covariance estimate with sample overlap correction #

SE ρ_g_: standard error of the estimate of ρ_g_

P: p-value from the statistical test for genetic covariance

P corrected: p-value from the statistical test for genetic covariance with sample overlap correction #

r_g_: genetic correlation estimate

r_g_ corrected: genetic correlation estimate with sample overlap correction #

h^2^_SNP_ 1: single-nucleotide polymorphism (SNP)-based heritability estimate for the first phenotype

h^2^_SNP_ 2: single-nucleotide polymorphism (SNP)-based heritability estimate for the second phenotype

# Anorexia nervosa sample overlapping for the UK Biobank cohort with the obesity, metabolic syndrome and type 2 diabetes mellitus sample

§ Note: the heritability values derived from annotation stratified analyses may be sometimes negative, or very small. The noise in the estimation of single-nucleotide polymorphism (SNP)-based heritability may cause the genetic correlation estimates to be out of bounds (>1) or even be set as missing (NA) when the heritability estimates are small or below zero, respectively. In such cases, genetic covariance estimates are less biased than genetic correlation measures.

# **Table S4 (c).** Results of the genetic covariance analyses between anorexia nervosa and type 2 diabetes mellitus stratified by insulin signalling gene-sets.

* Nominally significant stratified genetic covariance estimates (P < 0.05)

** Statistically significant stratified genetic covariance estimates (P < 2.06x10-4)

| *Gene-set name* | *n genes/gene-set* | *ρ_g_ corrected* | *SE ρ_g_* | *P corrected* | *r_g_ corrected* | *h^2^_SNP_ 1* | *h^2^_SNP_ 2* | *annotated SNPs* | *total SNPs* |
| --- | --- | --- | --- | --- | --- | --- | --- | --- | --- |
| BIOCARTA INSULIN PATHWAY | **21** | **-0.00042** | **0.00010** | **1.26x10-5 **** | **-0.67653** | **0.00078** | **0.00050** | **1,268** | **860,288** |
| KEGG INSULIN SIGNALING PATHWAY | 137 | -0.00109 | 0.00035 | 0.00171 * | -0.51725 | 0.00200 | 0.00222 | 9,953 | 860,288 |
| PID INSULIN PATHWAY | 44 | 0.00017 | 0.00017 | 0.32151 | 0.13338 | 0.00164 | 0.00103 | 3,894 | 860,288 |
| REACTOME INSULIN PROCESSING | **27** | **0.00059** | **0.00014** | **3.77x10-5 **** | **0.32217** | **0.00216** | **0.00153** | **2,742** | **860,288** |
| REACTOME INSULIN RECEPTOR RECYCLING | 26 | -0.00037 | 0.00011 | 0.00111 * | § NA | -0.00021 | 0.00119 | 1,823 | 860,288 |
| REACTOME INSULIN RECEPTOR SIGNALLING CASCADE | 54 | 0.00001 | 0.00018 | 0.97716 | 0.01330 | 0.00009 | 0.00169 | 3,749 | 860,288 |
| REACTOME REGULATION OF INSULIN SECRETION | 77 | -0.00104 | 0.00044 | 0.01914 * | -0.36360 | 0.00350 | 0.00234 | 8,624 | 860,288 |
| REACTOME SIGNALING BY INSULIN RECEPTOR | 78 | -0.00035 | 0.00024 | 0.14787 | -0.62668 | 0.00014 | 0.00229 | 5,427 | 860,288 |
| van de Vondervoort's gene-set | 53 | -0.00077 | 0.00033 | 0.02035 * | -0.29205 | 0.00292 | 0.00239 | 8,092 | 860,288 |

*Abbreviations*

ρ_g_: genetic covariance estimate

ρ_g_ corrected: genetic covariance estimate with sample overlap correction #

SE ρ_g_: standard error of the estimate of ρ_g_

P: p-value from the statistical test for genetic covariance

P corrected: p-value from the statistical test for genetic covariance with sample overlap correction #

r_g_: genetic correlation estimate

r_g_ corrected: genetic correlation estimate with sample overlap correction #

h^2^_SNP_ 1: single-nucleotide polymorphism (SNP)-based heritability estimate for the first phenotype

h^2^_SNP_ 2: single-nucleotide polymorphism (SNP)-based heritability estimate for the second phenotype

# Anorexia nervosa sample overlapping for the UK Biobank cohort with the obesity, metabolic syndrome and type 2 diabetes mellitus sample

§ Note: the heritability values derived from annotation stratified analyses may be sometimes negative, or very small. The noise in the estimation of single-nucleotide polymorphism (SNP)-based heritability may cause the genetic correlation estimates to be out of bounds (>1) or even be set as missing (NA) when the heritability estimates are small or below zero, respectively. In such cases, genetic covariance estimates are less biased than genetic correlation measures.

| **Table S5 (a).** Results of the genetic covariance analyses between attention-deficit/hyperactivity disorder and metabolic syndrome stratified by insulin signalling gene-sets. |
| --- |
| * Nominally significant stratified genetic covariance estimates (P < 0.05)  ** Statistically significant stratified genetic covariance estimates (P < 2.06x10-4) |

| *Gene-set name* | *n genes/gene-set* | *ρ_g_* | *SE ρ_g_* | *P* | *r_g_* | *h^2^_SNP_ 1* | *h^2^_SNP_ 2* | *annotated SNPs* | *total SNPs* |
| --- | --- | --- | --- | --- | --- | --- | --- | --- | --- |
| BIOCARTA INSULIN PATHWAY | 21 | 0.00020 | 0.00010 | 0.04856 * | 0.34669 | 0.00048 | 0.00067 | 1,552 | 986,120 |
| KEGG INSULIN SIGNALING PATHWAY | 137 | 0.00028 | 0.00045 | 0.54037 | 0.10965 | 0.00304 | 0.00211 | 11,571 | 986,120 |
| PID INSULIN PATHWAY | 44 | 0.00000 | 0.00025 | 0.99674 | § NA | -0.00054 | 0.00102 | 4,416 | 986,120 |
| REACTOME INSULIN PROCESSING | 27 | -0.00010 | 0.00015 | 0.49243 | § NA | -0.00014 | 0.00091 | 3,145 | 986,120 |
| REACTOME INSULIN RECEPTOR RECYCLING | 26 | 0.00019 | 0.00010 | 0.06650 | 0.23282 | 0.00100 | 0.00067 | 2,042 | 986,120 |
| REACTOME INSULIN RECEPTOR SIGNALLING CASCADE | 54 | 0.00051 | 0.00022 | 0.01715 * | § NA | -0.00072 | 0.00092 | 4,376 | 986,120 |
| REACTOME REGULATION OF INSULIN SECRETION | **77** | **0.00174** | **0.00045** | **0.00012 **** | **0.82293** | **0.00287** | **0.00156** | **9,850** | **986,120** |
| REACTOME SIGNALING BY INSULIN RECEPTOR | 78 | 0.00072 | 0.00027 | 0.00861 * | 1.02771 | 0.00035 | 0.00141 | 6,264 | 986,120 |
| van de Vondervoort's gene-set | 53 | 0.00140 | 0.00043 | 0.00106 * | 1.68137 | 0.00086 | 0.00081 | 9,061 | 986,120 |

*Abbreviations*

ρ_g_: genetic covariance estimate

SE ρ_g_: standard error of the estimate of ρ_g_

P: p-value from the statistical test for genetic covariance

r_g_: genetic correlation estimate

h^2^_SNP_ 1: single-nucleotide polymorphism (SNP)-based heritability estimate for the first phenotype

h^2^_SNP_ 2: single-nucleotide polymorphism (SNP)-based heritability estimate for the second phenotype

§ Note: the heritability values derived from annotation stratified analyses may be sometimes negative, or very small. The noise in the estimation of single-nucleotide polymorphism (SNP)-based heritability may cause the genetic correlation estimates to be out of bounds (>1) or even be set as missing (NA) when the heritability estimates are small or below zero, respectively. In such cases, genetic covariance estimates are less biased than genetic correlation measures.

# **Table S5 (b).** Results of the genetic covariance analyses between attention-deficit/hyperactivity disorder and obesity stratified by insulin signalling gene-sets.

* Nominally significant stratified genetic covariance estimates (P < 0.05)

** Statistically significant stratified genetic covariance estimates (P < 2.06x10-4)

| *Gene-set name* | *n genes/gene-set* | *ρ_g_* | *SE ρ_g_* | *P* | *r_g_* | *h^2^_SNP_ 1* | *h^2^_SNP_ 2* | *annotated SNPs* | *total SNPs* |
| --- | --- | --- | --- | --- | --- | --- | --- | --- | --- |
| BIOCARTA INSULIN PATHWAY | 21 | 0.00033 | 0.00012 | 0.00445 * | 0.80805 | 0.00049 | 0.00034 | 1,517 | 963,621 |
| KEGG INSULIN SIGNALING PATHWAY | 137 | 0.00218 | 0.00077 | 0.00438 * | 0.92186 | 0.00304 | 0.00184 | 11,307 | 963,621 |
| PID INSULIN PATHWAY | 44 | 0.00027 | 0.00028 | 0.32805 | § NA | -0.00051 | 0.00067 | 4,312 | 963,621 |
| REACTOME INSULIN PROCESSING | 27 | 0.00004 | 0.00017 | 0.83223 | 0.14993 | -0.00012 | -0.00052 | 3,093 | 963,621 |
| REACTOME INSULIN RECEPTOR RECYCLING | 26 | 0.00048 | 0.00021 | 0.02127 * | § NA | 0.00103 | -0.00026 | 1,989 | 963,621 |
| REACTOME INSULIN RECEPTOR SIGNALLING CASCADE | 54 | 0.00081 | 0.00036 | 0.02327 * | § NA | -0.00067 | 0.00317 | 4,272 | 963,621 |
| REACTOME REGULATION OF INSULIN SECRETION | 77 | 0.00209 | 0.00081 | 0.00966 * | § 1.21152 | 0.00300 | 0.00099 | 9,572 | 963,621 |
| REACTOME SIGNALING BY INSULIN RECEPTOR | 78 | 0.00127 | 0.00047 | 0.00639 * | § 1.10834 | 0.00043 | 0.00306 | 6,113 | 963,621 |
| van de Vondervoort's gene-set | 53 | 0.00136 | 0.00056 | 0.01483 * | § NA | 0.00071 | -0.00009 | 8,684 | 963,621 |

*Abbreviations*

ρ_g_: genetic covariance estimate

SE ρ_g_: standard error of the estimate of ρ_g_

P: p-value from the statistical test for genetic covariance

r_g_: genetic correlation estimate

h^2^_SNP_ 1: single-nucleotide polymorphism (SNP)-based heritability estimate for the first phenotype

h^2^_SNP_ 2: single-nucleotide polymorphism (SNP)-based heritability estimate for the second phenotype

§ Note: the heritability values derived from annotation stratified analyses may be sometimes negative, or very small. The noise in the estimation of single-nucleotide polymorphism (SNP)-based heritability may cause the genetic correlation estimates to be out of bounds (>1) or even be set as missing (NA) when the heritability estimates are small or below zero, respectively. In such cases, genetic covariance estimates are less biased than genetic correlation measures.

| **Table S5 (c).** Results of the genetic covariance analyses between attention-deficit/hyperactivity disorder and type 2 diabetes mellitus stratified by insulin signalling gene-sets. |
| --- |
| * Nominally significant stratified genetic covariance estimates (P < 0.05)  ** Statistically significant stratified genetic covariance estimates (P < 2.06x10-4) |

| *Gene-set name* | *n genes/gene-set* | *ρ_g_* | *SE ρg* | *P* | *r_g_* | *h^2^_SNP_ 1* | *h^2^_SNP_ 2* | *annotated SNPs* | *total SNPs* |
| --- | --- | --- | --- | --- | --- | --- | --- | --- | --- |
| BIOCARTA INSULIN PATHWAY | 21 | 0.00004 | 0.00008 | 0.62016 | 0.08005 | 0.00047 | 0.00053 | 1,552 | 985,453 |
| KEGG INSULIN SIGNALING PATHWAY | 137 | 0.00037 | 0.00047 | 0.43807 | 0.14243 | 0.00306 | 0.00218 | 11,556 | 985,453 |
| PID INSULIN PATHWAY | 44 | 0.00033 | 0.00015 | 0.02759 * | § NA | -0.00054 | 0.00093 | 4,413 | 985,453 |
| REACTOME INSULIN PROCESSING | 27 | 0.00024 | 0.00012 | 0.03793 * | § NA | -0.00015 | 0.00141 | 3,143 | 985,453 |
| REACTOME INSULIN RECEPTOR RECYCLING | 26 | -0.00020 | 0.00009 | 0.02283 * | -0.19259 | 0.00100 | 0.00103 | 2,039 | 985,453 |
| REACTOME INSULIN RECEPTOR SIGNALLING CASCADE | 54 | -0.00029 | 0.00016 | 0.07754 | § NA | -0.00072 | 0.00162 | 4,372 | 985,453 |
| REACTOME REGULATION OF INSULIN SECRETION | 77 | 0.00117 | 0.00042 | 0.00554 * | 0.47819 | 0.00289 | 0.00208 | 9,839 | 985,453 |
| REACTOME SIGNALING BY INSULIN RECEPTOR | 78 | -0.00046 | 0.00021 | 0.02680 * | -0.52113 | 0.00035 | 0.00226 | 6,258 | 985,453 |
| van de Vondervoort's gene-set | 53 | 0.00066 | 0.00031 | 0.03003 * | 0.48857 | 0.00087 | 0.00211 | 9,048 | 985,453 |

*Abbreviations*

ρ_g_: genetic covariance estimate

SE ρ_g_: standard error of the estimate of ρ_g_

P: p-value from the statistical test for genetic covariance

r_g_: genetic correlation estimate

h^2^_SNP_ 1: single-nucleotide polymorphism (SNP)-based heritability estimate for the first phenotype

h^2^_SNP_ 2: single-nucleotide polymorphism (SNP)-based heritability estimate for the second phenotype

§ Note: the heritability values derived from annotation stratified analyses may be sometimes negative, or very small. The noise in the estimation of single-nucleotide polymorphism (SNP)-based heritability may cause the genetic correlation estimates to be out of bounds (>1) or even be set as missing (NA) when the heritability estimates are small or below zero, respectively. In such cases, genetic covariance estimates are less biased than genetic correlation measures.

# **Table S6 (a).** Results of the genetic covariance analyses between autism spectrum disorder and metabolic syndrome stratified by insulin signalling gene-sets.

* Nominally significant stratified genetic covariance estimates (P < 0.05)

** Statistically significant stratified genetic covariance estimates (P < 2.06x10-4)

| \| *Gene-set name* \| *n genes/gene-set* \| *ρ_g_* \| *SE ρ_g_* \| *P* \| *r_g_* \| *h^2^_SNP_ 1* \| *h^2^_SNP_ 2* \| *annotated SNPs* \| *total SNPs* \| \| --- \| --- \| --- \| --- \| --- \| --- \| --- \| --- \| --- \| --- \| \| BIOCARTA INSULIN PATHWAY \| **21** \| **-0.00041** \| **0.00010** \| **1.96x10-5 **** \| **-0.72714** \| **0.00046** \| **0.00068** \| **1,520** \| **968,964** \| \| KEGG INSULIN SIGNALING PATHWAY \| **137** \| **-0.00170** \| **0.00041** \| **3.22x10-5 **** \| **-0.73026** \| **0.00261** \| **0.00207** \| **11,334** \| **968,964** \| \| PID INSULIN PATHWAY \| **44** \| **-0.00080** \| **0.00018** \| **1.25x10-5 **** \| **§ -2.2516** \| **0.00012** \| **0.00105** \| **4,319** \| **968,964** \| \| REACTOME INSULIN PROCESSING \| 27 \| -0.00042 \| 0.00018 \| 0.01727 * \| -0.46464 \| 0.00090 \| 0.00091 \| 3,096 \| 968,964 \| \| REACTOME INSULIN RECEPTOR RECYCLING \| 26 \| -0.00020 \| 0.00008 \| 0.01582 * \| § -1.6900 \| 0.00002 \| 0.00061 \| 1,998 \| 968,964 \| \| REACTOME INSULIN RECEPTOR SIGNALLING CASCADE \| 54 \| -0.00011 \| 0.00024 \| 0.65456 \| -0.09718 \| 0.00133 \| 0.00094 \| 4,277 \| 968,964 \| \| REACTOME REGULATION OF INSULIN SECRETION \| 77 \| 0.00088 \| 0.00038 \| 0.02061 * \| 0.58588 \| 0.00150 \| 0.00151 \| 9,642 \| 968,964 \| \| REACTOME SIGNALING BY INSULIN RECEPTOR \| 78 \| -0.00026 \| 0.00030 \| 0.38936 \| -0.19121 \| 0.00132 \| 0.00138 \| 6,131 \| 968,964 \| \| van de Vondervoort's gene-set \| 53 \| 0.00001 \| 0.00034 \| 0.96892 \| 0.06082 \| 0.00006 \| 0.00079 \| 8,815 \| 968,964 \| |
| --- | --- | --- | --- | --- | --- | --- | --- | --- | --- | --- | --- | --- | --- | --- | --- | --- | --- | --- | --- | --- | --- | --- | --- | --- | --- | --- | --- | --- | --- | --- | --- | --- | --- | --- | --- | --- | --- | --- | --- | --- | --- | --- | --- | --- | --- | --- | --- | --- | --- | --- | --- | --- | --- | --- | --- | --- | --- | --- | --- | --- | --- | --- | --- | --- | --- | --- | --- | --- | --- | --- | --- | --- | --- | --- | --- | --- | --- | --- | --- | --- | --- | --- | --- | --- | --- | --- | --- | --- | --- | --- | --- | --- | --- | --- | --- | --- | --- | --- | --- | --- |
|  |

*Abbreviations*

ρ_g_: genetic covariance estimate

SE ρ_g_: standard error of the estimate of ρ_g_

P: p-value from the statistical test for genetic covariance

r_g_: genetic correlation estimate

h^2^_SNP_ 1: single-nucleotide polymorphism (SNP)-based heritability estimate for the first phenotype

h^2^_SNP_ 2: single-nucleotide polymorphism (SNP)-based heritability estimate for the second phenotype

§ Note: the heritability values derived from annotation stratified analyses may be sometimes negative, or very small. The noise in the estimation of single-nucleotide polymorphism (SNP)-based heritability may cause the genetic correlation estimates to be out of bounds (>1) or even be set as missing (NA) when the heritability estimates are small or below zero, respectively. In such cases, genetic covariance estimates are less biased than genetic correlation measures.

# **Table S6 (b).** Results of the genetic covariance analyses between autism spectrum disorder and obesity stratified by insulin signalling gene-sets.

* Nominally significant stratified genetic covariance estimates (P < 0.05)

** Statistically significant stratified genetic covariance estimates (P < 2.06x10-4)

| \| *Gene-set name* \| *n genes/gene-set* \| *ρ_g_* \| *SE ρ_g_* \| *P* \| *r_g_* \| *h^2^_SNP_ 1* \| *h^2^_SNP_ 2* \| *annotated SNPs* \| *total SNPs* \| \| --- \| --- \| --- \| --- \| --- \| --- \| --- \| --- \| --- \| --- \| \| BIOCARTA INSULIN PATHWAY \| 21 \| -0.00010 \| 0.00014 \| 0.49473 \| -0.24325 \| 0.00047 \| 0.00034 \| 1,488 \| 948,627 \| \| KEGG INSULIN SIGNALING PATHWAY \| 137 \| -0.00047 \| 0.00075 \| 0.53570 \| -0.21682 \| 0.00253 \| 0.00183 \| 11,092 \| 948,627 \| \| PID INSULIN PATHWAY \| 44 \| -0.00029 \| 0.00024 \| 0.22168 \| -0.98940 \| 0.00013 \| 0.00067 \| 4,223 \| 948,627 \| \| REACTOME INSULIN PROCESSING \| 27 \| -0.00027 \| 0.00018 \| 0.13547 \| § NA \| 0.00090 \| -0.00049 \| 3,048 \| 948,627 \| \| REACTOME INSULIN RECEPTOR RECYCLING \| 26 \| -0.00032 \| 0.00014 \| 0.02266 * \| § NA \| 0.00003 \| -0.00023 \| 1,950 \| 948,627 \| \| REACTOME INSULIN RECEPTOR SIGNALLING CASCADE \| 54 \| -0.00018 \| 0.00040 \| 0.65960 \| -0.08605 \| 0.00134 \| 0.00317 \| 4,182 \| 948,627 \| \| REACTOME REGULATION OF INSULIN SECRETION \| 77 \| -0.00023 \| 0.00055 \| 0.68078 \| -0.17974 \| 0.00155 \| 0.00103 \| 9,394 \| 948,627 \| \| REACTOME SIGNALING BY INSULIN RECEPTOR \| 78 \| -0.00043 \| 0.00048 \| 0.37403 \| -0.20884 \| 0.00136 \| 0.00308 \| 5,993 \| 948,627 \| \| van de Vondervoort's gene-set \| 53 \| -0.00021 \| 0.00046 \| 0.64384 \| § NA \| 0.00004 \| -0.00013 \| 8,478 \| 948,627 \| |
| --- | --- | --- | --- | --- | --- | --- | --- | --- | --- | --- | --- | --- | --- | --- | --- | --- | --- | --- | --- | --- | --- | --- | --- | --- | --- | --- | --- | --- | --- | --- | --- | --- | --- | --- | --- | --- | --- | --- | --- | --- | --- | --- | --- | --- | --- | --- | --- | --- | --- | --- | --- | --- | --- | --- | --- | --- | --- | --- | --- | --- | --- | --- | --- | --- | --- | --- | --- | --- | --- | --- | --- | --- | --- | --- | --- | --- | --- | --- | --- | --- | --- | --- | --- | --- | --- | --- | --- | --- | --- | --- | --- | --- | --- | --- | --- | --- | --- | --- | --- | --- |
|  |

*Abbreviations*

ρ_g_: genetic covariance estimate

SE ρ_g_: standard error of the estimate of ρ_g_

P: p-value from the statistical test for genetic covariance

r_g_: genetic correlation estimate

h^2^_SNP_ 1: single-nucleotide polymorphism (SNP)-based heritability estimate for the first phenotype

h^2^_SNP_ 2: single-nucleotide polymorphism (SNP)-based heritability estimate for the second phenotype

§ Note: the heritability values derived from annotation stratified analyses may be sometimes negative, or very small. The noise in the estimation of single-nucleotide polymorphism (SNP)-based heritability may cause the genetic correlation estimates to be out of bounds (>1) or even be set as missing (NA) when the heritability estimates are small or below zero, respectively. In such cases, genetic covariance estimates are less biased than genetic correlation measures.

| \| **Table S6 (c).** Results of the genetic covariance analyses between autism spectrum disorder and type 2 diabetes mellitus stratified by insulin signalling gene-sets. * Nominally significant stratified genetic covariance estimates (P < 0.05)  ** Statistically significant stratified genetic covariance estimates (P < 2.06x10-4) \| \| --- \| \| \| *Gene-set name* \| *n genes/gene-set* \| *ρ_g_* \| *SE ρ_g_* \| *P* \| *r_g_* \| *h^2^_SNP_ 1* \| *h^2^_SNP_ 2* \| *annotated SNPs* \| *total SNPs* \| \| --- \| --- \| --- \| --- \| --- \| --- \| --- \| --- \| --- \| --- \| \| BIOCARTA INSULIN PATHWAY \| 21 \| -0.00004 \| 0.00008 \| 0.62341 \| -0.0825 \| 0.00047 \| 0.00052 \| 1,520 \| 968,306 \| \| KEGG INSULIN SIGNALING PATHWAY \| 137 \| -0.00188 \| 0.00064 \| 0.00344 * \| -0.7915 \| 0.00265 \| 0.00213 \| 11,319 \| 968,306 \| \| PID INSULIN PATHWAY \| 44 \| -0.00039 \| 0.00015 \| 0.00873 * \| § -1.1441 \| 0.00013 \| 0.00090 \| 4,316 \| 968,306 \| \| REACTOME INSULIN PROCESSING \| 27 \| -0.00050 \| 0.00014 \| 0.00028 * \| -0.4458 \| 0.00090 \| 0.00137 \| 3,094 \| 968,306 \| \| REACTOME INSULIN RECEPTOR RECYCLING \| 26 \| 0.00005 \| 0.00011 \| 0.61515 \| 0.2728 \| 0.00004 \| 0.00101 \| 1,995 \| 968,306 \| \| REACTOME INSULIN RECEPTOR SIGNALLING CASCADE \| 54 \| -0.00039 \| 0.00019 \| 0.04673 * \| -0.2632 \| 0.00134 \| 0.00162 \| 4,273 \| 968,306 \| \| REACTOME REGULATION OF INSULIN SECRETION \| 77 \| -0.00044 \| 0.00036 \| 0.22018 \| -0.2536 \| 0.00149 \| 0.00206 \| 9,631 \| 968,306 \| \| REACTOME SIGNALING BY INSULIN RECEPTOR \| 78 \| -0.00034 \| 0.00026 \| 0.18536 \| -0.1950 \| 0.00133 \| 0.00226 \| 6,125 \| 968,306 \| \| van de Vondervoort's gene-set \| 53 \| -0.00046 \| 0.00028 \| 0.10153 \| § -1.2757 \| 0.00007 \| 0.00193 \| 8,802 \| 968,306 \| \|   *Abbreviations*  ρ_g_: genetic covariance estimate  SE ρ_g_: standard error of the estimate of ρ_g_  P: p-value from the statistical test for genetic covariance  r_g_: genetic correlation estimate  h^2^_SNP_ 1: single-nucleotide polymorphism (SNP)-based heritability estimate for the first phenotype  h^2^_SNP_ 2: single-nucleotide polymorphism (SNP)-based heritability estimate for the second phenotype  § Note: the heritability values derived from annotation stratified analyses may be sometimes negative, or very small. The noise in the estimation of single-nucleotide polymorphism (SNP)-based heritability may cause the genetic correlation estimates to be out of bounds (>1) or even be set as missing (NA) when the heritability estimates are small or below zero, respectively. In such cases, genetic covariance estimates are less biased than genetic correlation measures. |
| --- | --- | --- | --- | --- | --- | --- | --- | --- | --- | --- | --- | --- | --- | --- | --- | --- | --- | --- | --- | --- | --- | --- | --- | --- | --- | --- | --- | --- | --- | --- | --- | --- | --- | --- | --- | --- | --- | --- | --- | --- | --- | --- | --- | --- | --- | --- | --- | --- | --- | --- | --- | --- | --- | --- | --- | --- | --- | --- | --- | --- | --- | --- | --- | --- | --- | --- | --- | --- | --- | --- | --- | --- | --- | --- | --- | --- | --- | --- | --- | --- | --- | --- | --- | --- | --- | --- | --- | --- | --- | --- | --- | --- | --- | --- | --- | --- | --- | --- | --- | --- | --- | --- |
|  |

# **Table S7 (a).** Results of the genetic covariance analyses between bipolar disorder and metabolic syndrome stratified by insulin signalling gene-sets.

* Nominally significant stratified genetic covariance estimates (P < 0.05)

** Statistically significant stratified genetic covariance estimates (P < 2.06x10-4)

| *Gene-set name* | *n genes/gene-set* | *ρ_g_ corrected* | *SE ρ_g_* | *P corrected* | *r_g_ corrected* | *h^2^_SNP_ 1* | *h^2^_SNP_ 2* | *annotated SNPs* | *total SNPs* |
| --- | --- | --- | --- | --- | --- | --- | --- | --- | --- |
| BIOCARTA INSULIN PATHWAY | 21 | -0.00023 | 0.00009 | 0.01693 * | -0.32871 | 0.00070 | 0.00067 | 1,610 | 1,027,553 |
| KEGG INSULIN SIGNALING PATHWAY | 137 | -0.00082 | 0.00029 | 0.00435 * | -0.33662 | 0.00259 | 0.00230 | 11,944 | 1,027,553 |
| PID INSULIN PATHWAY | **44** | **-0.00076** | **0.00018** | **2.03x10-5 **** | **-0.98583** | **0.00054** | **0.00109** | **4,580** | **1,027,553** |
| REACTOME INSULIN PROCESSING | 27 | 0.00015 | 0.00009 | 0.09265 | 0.42334 | 0.00014 | 0.00094 | 3,274 | 1,027,553 |
| REACTOME INSULIN RECEPTOR RECYCLING | 26 | -0.00028 | 0.00011 | 0.01019 * | -0.32413 | 0.00100 | 0.00074 | 2,149 | 1,027,553 |
| REACTOME INSULIN RECEPTOR SIGNALLING CASCADE | 54 | -0.00094 | 0.00031 | 0.00273 * | -0.53128 | 0.00342 | 0.00092 | 4,561 | 1,027,553 |
| REACTOME REGULATION OF INSULIN SECRETION | 77 | -0.00140 | 0.00072 | 0.05251 | -0.34269 | 0.00996 | 0.00168 | 10,349 | 1,027,553 |
| REACTOME SIGNALING BY INSULIN RECEPTOR | 78 | -0.00113 | 0.00041 | 0.00522 * | -0.42806 | 0.00474 | 0.00148 | 6,532 | 1,027,553 |
| van de Vondervoort's gene-set | 53 | 0.00009 | 0.00049 | 0.85574 | 0.04690 | 0.00437 | 0.00082 | 9,602 | 1,027,553 |

*Abbreviations*

ρ_g_: genetic covariance estimate

ρ_g_ corrected: genetic covariance estimate with sample overlap correction #

SE ρ_g_: standard error of the estimate of ρ_g_

P: p-value from the statistical test for genetic covariance

P corrected: p-value from the statistical test for genetic covariance with sample overlap correction #

r_g_: genetic correlation estimate

r_g_ corrected: genetic correlation estimate with sample overlap correction #

h^2^_SNP_ 1: single-nucleotide polymorphism (SNP)-based heritability estimate for the first phenotype

h^2^_SNP_ 2: single-nucleotide polymorphism (SNP)-based heritability estimate for the second phenotype

# Bipolar disorder sample overlapping for the UK Biobank cohort with the obesity, metabolic syndrome and type 2 diabetes mellitus sample

§ Note: the heritability values derived from annotation stratified analyses may be sometimes negative, or very small. The noise in the estimation of single-nucleotide polymorphism (SNP)-based heritability may cause the genetic correlation estimates to be out of bounds (>1) or even be set as missing (NA) when the heritability estimates are small or below zero, respectively. In such cases, genetic covariance estimates are less biased than genetic correlation measures.

# **Table S7 (b).** Results of the genetic covariance analyses between bipolar disorder and obesity stratified by insulin signalling gene-sets.

* Nominally significant stratified genetic covariance estimates (P < 0.05)

** Statistically significant stratified genetic covariance estimates (P < 2.06x10-4)

| *Gene-set name* | *n genes/gene-set* | *ρ_g_ corrected* | *SE ρ_g_* | *P corrected* | *r_g_ corrected* | *h^2^_SNP_ 1* | *h^2^_SNP_ 2* | *annotated SNPs* | *total SNPs* |
| --- | --- | --- | --- | --- | --- | --- | --- | --- | --- |
| BIOCARTA INSULIN PATHWAY | 21 | -0.00042 | 0.00015 | 0.00420 * | -0.73539 | 0.00069 | 0.00048 | 1,569 | 997,284 |
| KEGG INSULIN SIGNALING PATHWAY | 137 | -0.00046 | 0.00054 | 0.39744 | -0.21138 | 0.00255 | 0.00183 | 11,624 | 997,284 |
| PID INSULIN PATHWAY | 44 | -0.00021 | 0.00020 | 0.30356 | -0.30856 | 0.00057 | 0.00081 | 4,462 | 997,284 |
| REACTOME INSULIN PROCESSING | 27 | 0.00042 | 0.00012 | 0.00048 * | § NA | 0.00015 | -0.00051 | 3,205 | 997,284 |
| REACTOME INSULIN RECEPTOR RECYCLING | 26 | 0.00042 | 0.00025 | 0.09717 | § NA | 0.00103 | -0.00031 | 2,079 | 997,284 |
| REACTOME INSULIN RECEPTOR SIGNALLING CASCADE | 54 | -0.00047 | 0.00066 | 0.47177 | -0.13985 | 0.00344 | 0.00331 | 4,429 | 997,284 |
| REACTOME REGULATION OF INSULIN SECRETION | 77 | -0.00167 | 0.00094 | 0.07707 | -0.50273 | 0.00962 | 0.00115 | 9,977 | 997,284 |
| REACTOME SIGNALING BY INSULIN RECEPTOR | 78 | -0.00008 | 0.00077 | 0.92094 | -0.01969 | 0.00479 | 0.00317 | 6,336 | 997,284 |
| van de Vondervoort's gene-set | 53 | 0.00063 | 0.00069 | 0.36634 | § NA | 0.00413 | -0.00023 | 9,149 | 997,284 |

*Abbreviations*

ρ_g_: genetic covariance estimate

ρ_g_ corrected: genetic covariance estimate with sample overlap correction #

SE ρ_g_: standard error of the estimate of ρ_g_

P: p-value from the statistical test for genetic covariance

P corrected: p-value from the statistical test for genetic covariance with sample overlap correction #

r_g_: genetic correlation estimate

r_g_ corrected: genetic correlation estimate with sample overlap correction #

h^2^_SNP_ 1: single-nucleotide polymorphism (SNP)-based heritability estimate for the first phenotype

h^2^_SNP_ 2: single-nucleotide polymorphism (SNP)-based heritability estimate for the second phenotype

# Bipolar disorder sample overlapping for the UK Biobank cohort with the obesity, metabolic syndrome and type 2 diabetes mellitus sample

§ Note: the heritability values derived from annotation stratified analyses may be sometimes negative, or very small. The noise in the estimation of single-nucleotide polymorphism (SNP)-based heritability may cause the genetic correlation estimates to be out of bounds (>1) or even be set as missing (NA) when the heritability estimates are small or below zero, respectively. In such cases, genetic covariance estimates are less biased than genetic correlation measures.

**Table S7 (c).** Results of the genetic covariance analyses between bipolar disorder and type 2 diabetes mellitus stratified by insulin signalling gene-sets.

* Nominally significant stratified genetic covariance estimates (P < 0.05)

** Statistically significant stratified genetic covariance estimates (P < 2.06x10-4)

| *Gene-set name* | *n genes/gene-set* | *ρ_g_ corrected* | *SE ρ_g_* | *P corrected* | *r_g_ corrected* | *h^2^_SNP_ 1* | *h^2^_SNP_ 2* | *annotated SNPs* | *total SNPs* |
| --- | --- | --- | --- | --- | --- | --- | --- | --- | --- |
| BIOCARTA INSULIN PATHWAY | 21 | -0.00013 | 0.00008 | 0.10165 | -0.21121 | 0.00070 | 0.00053 | 1,608 | 1,026,853 |
| KEGG INSULIN SIGNALING PATHWAY | 137 | -0.00009 | 0.00031 | 0.76383 | -0.03596 | 0.00259 | 0.00256 | 11,933 | 1,026,853 |
| PID INSULIN PATHWAY | **44** | **-0.00057** | **0.00013** | **9.6x10-6 **** | **-0.70105** | **0.00054** | **0.00121** | **4,575** | **1,026,853** |
| REACTOME INSULIN PROCESSING | 27 | 0.00026 | 0.00008 | 0.00154 * | 0.54011 | 0.00014 | 0.00166 | 3,273 | 1,026,853 |
| REACTOME INSULIN RECEPTOR RECYCLING | 26 | -0.00025 | 0.00013 | 0.05088 | -0.22856 | 0.00100 | 0.00125 | 2,146 | 1,026,853 |
| REACTOME INSULIN RECEPTOR SIGNALLING CASCADE | 54 | -0.00024 | 0.00023 | 0.30703 | -0.09477 | 0.00342 | 0.00181 | 4,556 | 1,026,853 |
| REACTOME REGULATION OF INSULIN SECRETION | 77 | -0.00124 | 0.00054 | 0.02067 * | -0.25558 | 0.00992 | 0.00237 | 10,340 | 1,026,853 |
| REACTOME SIGNALING BY INSULIN RECEPTOR | 78 | -0.00041 | 0.00030 | 0.17152 | -0.12147 | 0.00473 | 0.00244 | 6,525 | 1,026,853 |
| van de Vondervoort's gene-set | 53 | -0.00107 | 0.00046 | 0.01932 * | -0.31947 | 0.00436 | 0.00258 | 9,589 | 1,026,853 |

*Abbreviations*

ρ_g_: genetic covariance estimate

ρ_g_ corrected: genetic covariance estimate with sample overlap correction #

SE ρ_g_: standard error of the estimate of ρ_g_

P: p-value from the statistical test for genetic covariance

P corrected: p-value from the statistical test for genetic covariance with sample overlap correction #

r_g_: genetic correlation estimate

r_g_ corrected: genetic correlation estimate with sample overlap correction #

h^2^_SNP_ 1: single-nucleotide polymorphism (SNP)-based heritability estimate for the first phenotype

h^2^_SNP_ 2: single-nucleotide polymorphism (SNP)-based heritability estimate for the second phenotype

# Bipolar disorder sample overlapping for the UK Biobank cohort with the obesity, metabolic syndrome and type 2 diabetes mellitus sample

§ Note: the heritability values derived from annotation stratified analyses may be sometimes negative, or very small. The noise in the estimation of single-nucleotide polymorphism (SNP)-based heritability may cause the genetic correlation estimates to be out of bounds (>1) or even be set as missing (NA) when the heritability estimates are small or below zero, respectively. In such cases, genetic covariance estimates are less biased than genetic correlation measures.

# **Table S8 (a).** Results of the genetic covariance analyses between major depressive disorder and metabolic syndrome stratified by insulin signalling gene-sets.

* Nominally significant stratified genetic covariance estimates (P < 0.05)

** Statistically significant stratified genetic covariance estimates (P < 2.06x10-4)

| *Gene-set name* | *n genes/gene-set* | *ρ_g_ corrected* | *SE ρ_g_* | *P corrected* | *r_g_ corrected* | *h^2^_SNP_ 1* | *h^2^_SNP_ 2* | *annotated SNPs* | *total SNPs* |
| --- | --- | --- | --- | --- | --- | --- | --- | --- | --- |
| BIOCARTA INSULIN PATHWAY | 21 | 0.00002 | 0.00005 | 0.74814 | 0.05523 | 0.00013 | 0.00075 | 1,688 | 1,052,205 |
| KEGG INSULIN SIGNALING PATHWAY | 137 | -0.00012 | 0.00017 | 0.46738 | -0.16990 | 0.00022 | 0.00236 | 12,662 | 1,052,205 |
| PID INSULIN PATHWAY | 44 | -0.00017 | 0.00007 | 0.01181 * | § -1.30297 | 0.00002 | 0.00118 | 4,782 | 1,052,205 |
| REACTOME INSULIN PROCESSING | 27 | -0.00009 | 0.00005 | 0.08412 | -0.63046 | 0.00002 | 0.00099 | 3,329 | 1,052,205 |
| REACTOME INSULIN RECEPTOR RECYCLING | 26 | 0.00006 | 0.00010 | 0.53418 | 0.06689 | 0.00121 | 0.00077 | 2,201 | 1,052,205 |
| REACTOME INSULIN RECEPTOR SIGNALLING CASCADE | 54 | -0.00017 | 0.00024 | 0.48933 | § -1.44700 | 0.00001 | 0.00098 | 4,769 | 1,052,205 |
| REACTOME REGULATION OF INSULIN SECRETION | 77 | 0.00142 | 0.00047 | 0.00235 * | 0.58676 | 0.00326 | 0.00179 | 10,751 | 1,052,205 |
| REACTOME SIGNALING BY INSULIN RECEPTOR | 78 | -0.00007 | 0.00029 | 0.82008 | -0.05289 | 0.00100 | 0.00157 | 6,764 | 1,052,205 |
| van de Vondervoort's gene-set | 53 | -0.00036 | 0.00046 | 0.42616 | -0.20948 | 0.00339 | 0.00089 | 9,833 | 1,052,205 |

*Abbreviations*

ρ_g_: genetic covariance estimate

ρ_g_ corrected: genetic covariance estimate with sample overlap correction #

SE ρ_g_: standard error of the estimate of ρ_g_

P: p-value from the statistical test for genetic covariance

P corrected: p-value from the statistical test for genetic covariance with sample overlap correction #

r_g_: genetic correlation estimate

r_g_ corrected: genetic correlation estimate with sample overlap correction #

h^2^_SNP_ 1: single-nucleotide polymorphism (SNP)-based heritability estimate for the first phenotype

h^2^_SNP_ 2: single-nucleotide polymorphism (SNP)-based heritability estimate for the second phenotype

# Major depressive disorder sample overlapping for the UK Biobank cohort with the obesity, metabolic syndrome and type 2 diabetes mellitus sample

§ Note: the heritability values derived from annotation stratified analyses may be sometimes negative, or very small. The noise in the estimation of single-nucleotide polymorphism (SNP)-based heritability may cause the genetic correlation estimates to be out of bounds (>1) or even be set as missing (NA) when the heritability estimates are small or below zero, respectively. In such cases, genetic covariance estimates are less biased than genetic correlation measures.

# **Table S8 (b).** Results of the genetic covariance analyses between major depressive disorder and obesity stratified by insulin signalling gene-sets.

* Nominally significant stratified genetic covariance estimates (P < 0.05)

** Statistically significant stratified genetic covariance estimates (P < 2.06x10-4)

| *Gene-set name* | *n genes/gene-set* | *ρ_g_ corrected* | *SE ρ_g_* | *P corrected* | *r_g_ corrected* | *h^2^_SNP_ 1* | *h^2^_SNP_ 2* | *annotated SNPs* | *total SNPs* |
| --- | --- | --- | --- | --- | --- | --- | --- | --- | --- |
| BIOCARTA INSULIN PATHWAY | 21 | -0.00017 | 0.00008 | 0.03404 * | -0.63539 | 0.00013 | 0.00054 | 1,638 | 1,020,437 |
| KEGG INSULIN SIGNALING PATHWAY | 137 | 0.00007 | 0.00027 | 0.79991 | 0.10126 | 0.00022 | 0.00206 | 12,265 | 1,020,437 |
| PID INSULIN PATHWAY | 44 | -0.00014 | 0.00011 | 0.21876 | -0.97869 | 0.00002 | 0.00099 | 4,651 | 1,020,437 |
| REACTOME INSULIN PROCESSING | 27 | 0.00005 | 0.00007 | 0.42845 | § NA | 0.00002 | -0.00049 | 3,261 | 1,020,437 |
| REACTOME INSULIN RECEPTOR RECYCLING | 26 | 0.00019 | 0.00017 | 0.25685 | § NA | 0.00120 | -0.00032 | 2,128 | 1,020,437 |
| REACTOME INSULIN RECEPTOR SIGNALLING CASCADE | 54 | 0.00003 | 0.00048 | 0.95685 | § NA | -0.00003 | 0.00341 | 4,628 | 1,020,437 |
| REACTOME REGULATION OF INSULIN SECRETION | 77 | -0.00092 | 0.00069 | 0.17985 | -0.43397 | 0.00335 | 0.00134 | 10,332 | 1,020,437 |
| REACTOME SIGNALING BY INSULIN RECEPTOR | 78 | 0.00022 | 0.00058 | 0.70552 | 0.12467 | 0.00096 | 0.00325 | 6,562 | 1,020,437 |
| van de Vondervoort's gene-set | 53 | 0.00147 | 0.00069 | 0.03366 * | § NA | 0.00321 | -0.00010 | 9,395 | 1,020,437 |

*Abbreviations*

ρ_g_: genetic covariance estimate

ρ_g_ corrected: genetic covariance estimate with sample overlap correction #

SE ρ_g_: standard error of the estimate of ρ_g_

P: p-value from the statistical test for genetic covariance

P corrected: p-value from the statistical test for genetic covariance with sample overlap correction #

r_g_: genetic correlation estimate

r_g_ corrected: genetic correlation estimate with sample overlap correction #

h^2^_SNP_ 1: single-nucleotide polymorphism (SNP)-based heritability estimate for the first phenotype

h^2^_SNP_ 2: single-nucleotide polymorphism (SNP)-based heritability estimate for the second phenotype

# Major depressive disorder sample overlapping for the UK Biobank cohort with the obesity, metabolic syndrome and type 2 diabetes mellitus sample

§ Note: the heritability values derived from annotation stratified analyses may be sometimes negative, or very small. The noise in the estimation of single-nucleotide polymorphism (SNP)-based heritability may cause the genetic correlation estimates to be out of bounds (>1) or even be set as missing (NA) when the heritability estimates are small or below zero, respectively. In such cases, genetic covariance estimates are less biased than genetic correlation measures.

**Table S8 (c).** Results of the genetic covariance analyses between major depressive disorder and type 2 diabetes mellitus stratified by insulin signalling gene-sets.

* Nominally significant stratified genetic covariance estimates (P < 0.05)

** Statistically significant stratified genetic covariance estimates (P < 2.06x10-4)

| *Gene-set name* | *n genes/gene-set* | *ρ_g_ corrected* | *SE ρ_g_* | *P corrected* | *r_g_ corrected* | *h^2^_SNP_ 1* | *h^2^_SNP_ 2* | *annotated SNPs* | *total SNPs* |
| --- | --- | --- | --- | --- | --- | --- | --- | --- | --- |
| BIOCARTA INSULIN PATHWAY | 21 | 0.00002 | 0.00005 | 0.74089 | 0.06451 | 0.00013 | 0.00054 | 1,686 | 1,051,079 |
| KEGG INSULIN SIGNALING PATHWAY | 137 | -0.00006 | 0.00018 | 0.74373 | -0.07710 | 0.00022 | 0.00269 | 12,644 | 1,051,079 |
| PID INSULIN PATHWAY | 44 | -0.00019 | 0.00006 | 0.00175 * | § -1.36099 | 0.00001 | 0.00129 | 4,777 | 1,051,079 |
| REACTOME INSULIN PROCESSING | 27 | 0.00009 | 0.00004 | 0.05555 | 0.47321 | 0.00002 | 0.00176 | 3,327 | 1,051,079 |
| REACTOME INSULIN RECEPTOR RECYCLING | 26 | -0.00009 | 0.00013 | 0.49113 | -0.07366 | 0.00122 | 0.00129 | 2,197 | 1,051,079 |
| REACTOME INSULIN RECEPTOR SIGNALLING CASCADE | 54 | 0.00013 | 0.00024 | 0.59088 | § NA | 0.00000 | 0.00214 | 4,765 | 1,051,079 |
| REACTOME REGULATION OF INSULIN SECRETION | 77 | 0.00063 | 0.00045 | 0.16045 | 0.21871 | 0.00329 | 0.00251 | 10,740 | 1,051,079 |
| REACTOME SIGNALING BY INSULIN RECEPTOR | 78 | 0.00016 | 0.00029 | 0.58178 | 0.09693 | 0.00099 | 0.00276 | 6,757 | 1,051,079 |
| van de Vondervoort's gene-set | 53 | -0.00002 | 0.00037 | 0.96220 | -0.00574 | 0.00338 | 0.00271 | 9,820 | 1,051,079 |

*Abbreviations*

ρ_g_: genetic covariance estimate

ρ_g_ corrected: genetic covariance estimate with sample overlap correction #

SE ρ_g_: standard error of the estimate of ρ_g_

P: p-value from the statistical test for genetic covariance

P corrected: p-value from the statistical test for genetic covariance with sample overlap correction #

r_g_: genetic correlation estimate

r_g_ corrected: genetic correlation estimate with sample overlap correction #

h^2^_SNP_ 1: single-nucleotide polymorphism (SNP)-based heritability estimate for the first phenotype

h^2^_SNP_ 2: single-nucleotide polymorphism (SNP)-based heritability estimate for the second phenotype

# Major depressive disorder sample overlapping for the UK Biobank cohort with the obesity, metabolic syndrome and type 2 diabetes mellitus sample

§ Note: the heritability values derived from annotation stratified analyses may be sometimes negative, or very small. The noise in the estimation of single-nucleotide polymorphism (SNP)-based heritability may cause the genetic correlation estimates to be out of bounds (>1) or even be set as missing (NA) when the heritability estimates are small or below zero, respectively. In such cases, genetic covariance estimates are less biased than genetic correlation measures.

| **Table S9 (a).** Results of the genetic covariance analyses between obsessive-compulsive disorder and metabolic syndrome stratified by insulin signalling gene-sets. |
| --- |
| * Nominally significant stratified genetic covariance estimates (P < 0.05)  ** Statistically significant stratified genetic covariance estimates (P < 2.06x10-4) |

| *Gene-set name* | *n genes/gene-set* | *ρ_g_* | *SE ρ_g_* | *P* | *r_g_* | *h^2^_SNP_ 1* | *h^2^_SNP_ 2* | *annotated SNPs* | *total SNPs* |
| --- | --- | --- | --- | --- | --- | --- | --- | --- | --- |
| BIOCARTA INSULIN PATHWAY | 21 | -0.00090 | 0.00033 | 0.00569 * | -0.70027 | 0.00222 | 0.00075 | 1,612 | 1,019,413 |
| KEGG INSULIN SIGNALING PATHWAY | 137 | -0.00165 | 0.00123 | 0.17893 | -0.31180 | 0.01210 | 0.00231 | 12,069 | 1,019,413 |
| PID INSULIN PATHWAY | 44 | -0.00141 | 0.00053 | 0.00796 * | -0.84847 | 0.00244 | 0.00113 | 4,582 | 1,019,413 |
| REACTOME INSULIN PROCESSING | 27 | -0.00070 | 0.00030 | 0.02074 * | § NA | -0.00280 | 0.00094 | 3,216 | 1,019,413 |
| REACTOME INSULIN RECEPTOR RECYCLING | **26** | **-0.00124** | **0.00028** | **7.5x10-6 **** | **-0.81380** | **0.00316** | **0.00074** | **2,132** | **1,019,413** |
| REACTOME INSULIN RECEPTOR SIGNALLING CASCADE | 54 | -0.00046 | 0.00059 | 0.43263 | -0.24860 | 0.00362 | 0.00095 | 4,602 | 1,019,413 |
| REACTOME REGULATION OF INSULIN SECRETION | 77 | -0.00133 | 0.00077 | 0.08607 | -0.55682 | 0.00322 | 0.00176 | 10,345 | 1,019,413 |
| REACTOME SIGNALING BY INSULIN RECEPTOR | 78 | -0.00155 | 0.00073 | 0.03500 * | -0.49421 | 0.00646 | 0.00151 | 6,553 | 1,019,413 |

*Abbreviations*

ρ_g_: genetic covariance estimate

SE ρ_g_: standard error of the estimate of ρ_g_

P: p-value from the statistical test for genetic covariance

r_g_: genetic correlation estimate

h^2^_SNP_ 1: single-nucleotide polymorphism (SNP)-based heritability estimate for the first phenotype

h^2^_SNP_ 2: single-nucleotide polymorphism (SNP)-based heritability estimate for the second phenotype

§ Note: the heritability values derived from annotation stratified analyses may be sometimes negative, or very small. The noise in the estimation of single-nucleotide polymorphism (SNP)-based heritability may cause the genetic correlation estimates to be out of bounds (>1) or even be set as missing (NA) when the heritability estimates are small or below zero, respectively. In such cases, genetic covariance estimates are less biased than genetic correlation measures.

# **Table S9 (b).** Results of the genetic covariance analyses between obsessive-compulsive disorder and obesity stratified by insulin signalling gene-sets.

* Nominally significant stratified genetic covariance estimates (P < 0.05)

** Statistically significant stratified genetic covariance estimates (P < 2.06x10-4)

| *Gene-set name* | *n genes/gene-set* | *ρ_g_* | *SE ρ_g_* | *P* | *r_g_* | *h^2^_SNP_ 1* | *h^2^_SNP_ 2* | *annotated SNPs* | *total SNPs* |
| --- | --- | --- | --- | --- | --- | --- | --- | --- | --- |
| BIOCARTA INSULIN PATHWAY | 21 | -0.00102 | 0.00048 | 0.03299 * | -0.95125 | 0.00238 | 0.00049 | 1,558 | 988,989 |
| KEGG INSULIN SIGNALING PATHWAY | 137 | -0.00325 | 0.00178 | 0.06796 | -0.64832 | 0.01241 | 0.00203 | 11,680 | 988,989 |
| PID INSULIN PATHWAY | 44 | -0.00172 | 0.00066 | 0.00894 * | § -1.0861 | 0.00271 | 0.00092 | 4,435 | 988,989 |
| REACTOME INSULIN PROCESSING | 27 | -0.00040 | 0.00036 | 0.27014 | -0.33931 | -0.00271 | -0.00050 | 3,157 | 988,989 |
| REACTOME INSULIN RECEPTOR RECYCLING | 26 | 0.00019 | 0.00048 | 0.68795 | § NA | 0.00293 | -0.00027 | 2,055 | 988,989 |
| REACTOME INSULIN RECEPTOR SIGNALLING CASCADE | 54 | -0.00236 | 0.00098 | 0.01577 * | -0.70475 | 0.00345 | 0.00325 | 4,447 | 988,989 |
| REACTOME REGULATION OF INSULIN SECRETION | 77 | -0.00123 | 0.00136 | 0.36745 | -0.63540 | 0.00334 | 0.00112 | 9,967 | 988,989 |
| REACTOME SIGNALING BY INSULIN RECEPTOR | 78 | -0.00220 | 0.00128 | 0.08491 | -0.50419 | 0.00608 | 0.00313 | 6,329 | 988,989 |

*Abbreviations*

ρ_g_: genetic covariance estimate

SE ρ_g_: standard error of the estimate of ρ_g_

P: p-value from the statistical test for genetic covariance

r_g_: genetic correlation estimate

h^2^_SNP_ 1: single-nucleotide polymorphism (SNP)-based heritability estimate for the first phenotype

h^2^_SNP_ 2: single-nucleotide polymorphism (SNP)-based heritability estimate for the second phenotype

§ Note: the heritability values derived from annotation stratified analyses may be sometimes negative, or very small. The noise in the estimation of single-nucleotide polymorphism (SNP)-based heritability may cause the genetic correlation estimates to be out of bounds (>1) or even be set as missing (NA) when the heritability estimates are small or below zero, respectively. In such cases, genetic covariance estimates are less biased than genetic correlation measures.

| **Table S9 (c).** Results of the genetic covariance analyses between obsessive-compulsive disorder and type 2 diabetes mellitus stratified by insulin signalling gene-sets. |
| --- |
| * Nominally significant stratified genetic covariance estimates (P < 0.05)  ** Statistically significant stratified genetic covariance estimates (P < 2.06x10-4) |

| *Gene-set name* | *n genes/gene-set* | *ρ_g_* | *SE ρg* | *P* | *r_g_* | *h^2^_SNP_ 1* | *h^2^_SNP_ 2* | *annotated SNPs* | *total SNPs* |
| --- | --- | --- | --- | --- | --- | --- | --- | --- | --- |
| BIOCARTA INSULIN PATHWAY | 21 | -0.00044 | 0.00034 | 0.18916 | -0.4182 | 0.00221 | 0.00051 | 1,612 | 1,019,648 |
| KEGG INSULIN SIGNALING PATHWAY | 137 | -0.00012 | 0.00126 | 0.92503 | -0.0216 | 0.01205 | 0.00249 | 12,070 | 1,019,648 |
| PID INSULIN PATHWAY | 44 | -0.00046 | 0.00038 | 0.21967 | -0.2775 | 0.00231 | 0.00121 | 4,580 | 1,019,648 |
| REACTOME INSULIN PROCESSING | 27 | 0.00028 | 0.00023 | 0.21718 | § NA | -0.00284 | 0.00169 | 3,214 | 1,019,648 |
| REACTOME INSULIN RECEPTOR RECYCLING | **26** | **-0.00100** | **0.00026** | **0.00016 **** | **-0.5067** | **0.00304** | **0.00128** | **2,130** | **1,019,648** |
| REACTOME INSULIN RECEPTOR SIGNALLING CASCADE | 54 | -0.00051 | 0.00058 | 0.38055 | -0.1878 | 0.00365 | 0.00203 | 4,607 | 1,019,648 |
| REACTOME REGULATION OF INSULIN SECRETION | 77 | 0.00040 | 0.00071 | 0.57022 | 0.1435 | 0.00321 | 0.00248 | 10,344 | 1,019,648 |
| REACTOME SIGNALING BY INSULIN RECEPTOR | 78 | -0.00132 | 0.00072 | 0.06715 | -0.3171 | 0.00642 | 0.00268 | 6,557 | 1,019,648 |

*Abbreviations*

ρ_g_: genetic covariance estimate

SE ρ_g_: standard error of the estimate of ρ_g_

P: p-value from the statistical test for genetic covariance

r_g_: genetic correlation estimate

h^2^_SNP_ 1: single-nucleotide polymorphism (SNP)-based heritability estimate for the first phenotype

h^2^_SNP_ 2: single-nucleotide polymorphism (SNP)-based heritability estimate for the second phenotype

§ Note: the heritability values derived from annotation stratified analyses may be sometimes negative, or very small. The noise in the estimation of single-nucleotide polymorphism (SNP)-based heritability may cause the genetic correlation estimates to be out of bounds (>1) or even be set as missing (NA) when the heritability estimates are small or below zero, respectively. In such cases, genetic covariance estimates are less biased than genetic correlation measures.

| **Table S10 (a).** Results of the genetic covariance analyses between Tourette’s syndrome and metabolic syndrome stratified by insulin signalling gene-sets. |
| --- |
| * Nominally significant stratified genetic covariance estimates (P < 0.05)  ** Statistically significant stratified genetic covariance estimates (P < 2.06x10-4) |

| *Gene-set name* | *n genes/gene-set* | *ρ_g_* | *SE ρ_g_* | *P* | *r_g_* | *h^2^_SNP_ 1* | *h^2^_SNP_ 2* | *annotated SNPs* | *total SNPs* |
| --- | --- | --- | --- | --- | --- | --- | --- | --- | --- |
| BIOCARTA INSULIN PATHWAY | 21 | 0.00023 | 0.00022 | 0.29049 | § NA | -0.00051 | 0.00074 | 1,661 | 1,035,514 |
| KEGG INSULIN SIGNALING PATHWAY | 137 | 0.00033 | 0.00088 | 0.70622 | 0.09282 | 0.00527 | 0.00241 | 12,337 | 1,035,514 |
| PID INSULIN PATHWAY | 44 | 0.00060 | 0.00037 | 0.10536 | § 2.26205 | 0.00006 | 0.00114 | 4,666 | 1,035,514 |
| REACTOME INSULIN PROCESSING | 27 | 0.00000 | 0.00025 | 0.98879 | -0.00553 | 0.00040 | 0.00098 | 3,268 | 1,035,514 |
| REACTOME INSULIN RECEPTOR RECYCLING | 26 | -0.00006 | 0.00010 | 0.58767 | § NA | -0.00052 | 0.00076 | 2,172 | 1,035,514 |
| REACTOME INSULIN RECEPTOR SIGNALLING CASCADE | 54 | 0.00079 | 0.00021 | 0.00022 * | 0.70925 | 0.00131 | 0.00095 | 4,715 | 1,035,514 |
| REACTOME REGULATION OF INSULIN SECRETION | 77 | 0.00106 | 0.00039 | 0.00590 * | 0.97438 | 0.00067 | 0.00177 | 10,539 | 1,035,514 |
| REACTOME SIGNALING BY INSULIN RECEPTOR | 78 | 0.00066 | 0.00028 | 0.01959 * | 0.55058 | 0.00093 | 0.00153 | 6,686 | 1,035,514 |
| van de Vondervoort's gene-set | 53 | -0.00001 | 0.00034 | 0.97756 | -0.01048 | 0.00098 | 0.00086 | 9,789 | 1,035,514 |

*Abbreviations*

ρ_g_: genetic covariance estimate

SE ρ_g_: standard error of the estimate of ρ_g_

P: p-value from the statistical test for genetic covariance

r_g_: genetic correlation estimate

h^2^_SNP_ 1: single-nucleotide polymorphism (SNP)-based heritability estimate for the first phenotype

h^2^_SNP_ 2: single-nucleotide polymorphism (SNP)-based heritability estimate for the second phenotype

§ Note: the heritability values derived from annotation stratified analyses may be sometimes negative, or very small. The noise in the estimation of single-nucleotide polymorphism (SNP)-based heritability may cause the genetic correlation estimates to be out of bounds (>1) or even be set as missing (NA) when the heritability estimates are small or below zero, respectively. In such cases, genetic covariance estimates are less biased than genetic correlation measures.

# **Table S10 (b).** Results of the genetic covariance analyses between Tourette’s syndrome and obesity stratified by insulin signalling gene-sets.

* Nominally significant stratified genetic covariance estimates (P < 0.05)

** Statistically significant stratified genetic covariance estimates (P < 2.06x10-4)

| *Gene-set name* | *n genes/gene-set* | *ρ_g_* | *SE ρ_g_* | *P* | *r_g_* | *h^2^_SNP_ 1* | *h^2^_SNP_ 2* | *annotated SNPs* | *total SNPs* |
| --- | --- | --- | --- | --- | --- | --- | --- | --- | --- |
| BIOCARTA INSULIN PATHWAY | 21 | -0.00021 | 0.00025 | 0.40022 | § NA | -0.00045 | 0.00049 | 1,607 | 1,001,953 |
| KEGG INSULIN SIGNALING PATHWAY | 137 | -0.00319 | 0.00130 | 0.01379 * | -0.99394 | 0.00525 | 0.00197 | 11,941 | 1,001,953 |
| PID INSULIN PATHWAY | 44 | -0.00143 | 0.00053 | 0.00699 * | § NA | -0.00010 | 0.00089 | 4,522 | 1,001,953 |
| REACTOME INSULIN PROCESSING | 27 | 0.00106 | 0.00035 | 0.00272 * | § NA | 0.00034 | -0.00048 | 3,199 | 1,001,953 |
| REACTOME INSULIN RECEPTOR RECYCLING | 26 | 0.00023 | 0.00015 | 0.12917 | 0.59671 | -0.00050 | -0.00031 | 2,093 | 1,001,953 |
| REACTOME INSULIN RECEPTOR SIGNALLING CASCADE | 54 | -0.00021 | 0.00043 | 0.62190 | -0.10019 | 0.00130 | 0.00341 | 4,546 | 1,001,953 |
| REACTOME REGULATION OF INSULIN SECRETION | 77 | 0.00026 | 0.00055 | 0.64294 | 0.29736 | 0.00060 | 0.00124 | 10,118 | 1,001,953 |
| REACTOME SIGNALING BY INSULIN RECEPTOR | 78 | -0.00001 | 0.00055 | 0.98421 | -0.00624 | 0.00094 | 0.00325 | 6,448 | 1,001,953 |
| van de Vondervoort's gene-set | 53 | -0.00083 | 0.00056 | 0.14112 | § NA | 0.00106 | -0.00007 | 9,246 | 1,001,953 |

*Abbreviations*

ρ_g_: genetic covariance estimate

SE ρ_g_: standard error of the estimate of ρ_g_

P: p-value from the statistical test for genetic covariance

r_g_: genetic correlation estimate

h^2^_SNP_ 1: single-nucleotide polymorphism (SNP)-based heritability estimate for the first phenotype

h^2^_SNP_ 2: single-nucleotide polymorphism (SNP)-based heritability estimate for the second phenotype

§ Note: the heritability values derived from annotation stratified analyses may be sometimes negative, or very small. The noise in the estimation of single-nucleotide polymorphism (SNP)-based heritability may cause the genetic correlation estimates to be out of bounds (>1) or even be set as missing (NA) when the heritability estimates are small or below zero, respectively. In such cases, genetic covariance estimates are less biased than genetic correlation measures.

| **Table S10 (c).** Results of the genetic covariance analyses between Tourette’s syndrome and type 2 diabetes mellitus stratified by insulin signalling gene-sets. |
| --- |
| * Nominally significant stratified genetic covariance estimates (P < 0.05)  ** Statistically significant stratified genetic covariance estimates (P < 2.06x10-4) |

| *Gene-set name* | *n genes/gene-set* | *ρ_g_* | *SE ρg* | *P* | *r_g_* | *h^2^_SNP_ 1* | *h^2^_SNP_ 2* | *annotated SNPs* | *total SNPs* |
| --- | --- | --- | --- | --- | --- | --- | --- | --- | --- |
| BIOCARTA INSULIN PATHWAY | 21 | -0.00042 | 0.00014 | 0.00215 * | § NA | -0.00051 | 0.00055 | 1,661 | 1,035,646 |
| KEGG INSULIN SIGNALING PATHWAY | 137 | 0.00010 | 0.00073 | 0.89439 | 0.02565 | 0.00537 | 0.00266 | 12,337 | 1,035,646 |
| PID INSULIN PATHWAY | 44 | 0.00017 | 0.00026 | 0.51152 | 0.55905 | 0.00007 | 0.00125 | 4,664 | 1,035,646 |
| REACTOME INSULIN PROCESSING | 27 | -0.00043 | 0.00021 | 0.03975 * | -0.50855 | 0.00042 | 0.00171 | 3,266 | 1,035,646 |
| REACTOME INSULIN RECEPTOR RECYCLING | 26 | -0.00001 | 0.00010 | 0.94225 | § NA | -0.00052 | 0.00128 | 2,169 | 1,035,646 |
| REACTOME INSULIN RECEPTOR SIGNALLING CASCADE | 54 | 0.00049 | 0.00025 | 0.04385 * | 0.30201 | 0.00132 | 0.00203 | 4,719 | 1,035,646 |
| REACTOME REGULATION OF INSULIN SECRETION | 77 | 0.00030 | 0.00037 | 0.41855 | 0.22757 | 0.00069 | 0.00246 | 10,537 | 1,035,646 |
| REACTOME SIGNALING BY INSULIN RECEPTOR | 78 | 0.00043 | 0.00029 | 0.13306 | 0.27349 | 0.00094 | 0.00268 | 6,688 | 1,035,646 |
| van de Vondervoort's gene-set | 53 | -0.00042 | 0.00034 | 0.21149 | -0.25978 | 0.00096 | 0.00272 | 9,787 | 1,035,646 |

*Abbreviations*

ρ_g_: genetic covariance estimate

SE ρ_g_: standard error of the estimate of ρ_g_

P: p-value from the statistical test for genetic covariance

r_g_: genetic correlation estimate

h^2^_SNP_ 1: single-nucleotide polymorphism (SNP)-based heritability estimate for the first phenotype

h^2^_SNP_ 2: single-nucleotide polymorphism (SNP)-based heritability estimate for the second phenotype

§ Note: the heritability values derived from annotation stratified analyses may be sometimes negative, or very small. The noise in the estimation of single-nucleotide polymorphism (SNP)-based heritability may cause the genetic correlation estimates to be out of bounds (>1) or even be set as missing (NA) when the heritability estimates are small or below zero, respectively. In such cases, genetic covariance estimates are less biased than genetic correlation measures.

| **Table S11 (a).** Results of the genetic covariance analyses between schizophrenia and metabolic syndrome stratified by insulin signalling gene-sets. |
| --- |
| * Nominally significant stratified genetic covariance estimates (P < 0.05)  ** Statistically significant stratified genetic covariance estimates (P < 2.06x10-4) |

| *Gene-set name* | *n genes/gene-set* | *ρ_g_* | *SE ρ_g_* | *P* | *r_g_* | *h^2^_SNP_ 1* | *h^2^_SNP_ 2* | *annotated SNPs* | *total SNPs* |
| --- | --- | --- | --- | --- | --- | --- | --- | --- | --- |
| BIOCARTA INSULIN PATHWAY | 21 | -0.00026 | 0.00020 | 0.19088 | -0.23744 | 0.00158 | 0.00074 | 1,708 | 1,049,783 |
| KEGG INSULIN SIGNALING PATHWAY | 137 | -0.00086 | 0.00061 | 0.15658 | -0.23817 | 0.00547 | 0.00241 | 12,777 | 1,049,783 |
| PID INSULIN PATHWAY | 44 | -0.00141 | 0.00032 | 1.32x10-5 ** | § -1.05211 | 0.00155 | 0.00117 | 4,836 | 1,049,783 |
| REACTOME INSULIN PROCESSING | 27 | 0.00002 | 0.00014 | 0.88552 | 0.02150 | 0.00087 | 0.00100 | 3,371 | 1,049,783 |
| REACTOME INSULIN RECEPTOR RECYCLING | 26 | -0.00038 | 0.00016 | 0.01673 * | -0.25629 | 0.00290 | 0.00076 | 2,228 | 1,049,783 |
| REACTOME INSULIN RECEPTOR SIGNALLING CASCADE | 54 | -0.00111 | 0.00045 | 0.01368 * | -0.56355 | 0.00417 | 0.00094 | 4,877 | 1,049,783 |
| REACTOME REGULATION OF INSULIN SECRETION | 77 | -0.00154 | 0.00080 | 0.05474 * | -0.29646 | 0.01472 | 0.00184 | 10,848 | 1,049,783 |
| REACTOME SIGNALING BY INSULIN RECEPTOR | 78 | -0.00156 | 0.00056 | 0.00526 * | -0.46908 | 0.00718 | 0.00153 | 6,905 | 1,049,783 |
| van de Vondervoort's gene-set | 53 | -0.00041 | 0.00056 | 0.46475 | -0.18001 | 0.00592 | 0.00088 | 10,062 | 1,049,783 |

*Abbreviations*

ρ_g_: genetic covariance estimate

SE ρ_g_: standard error of the estimate of ρ_g_

P: p-value from the statistical test for genetic covariance

r_g_: genetic correlation estimate

h^2^_SNP_ 1: single-nucleotide polymorphism (SNP)-based heritability estimate for the first phenotype

h^2^_SNP_ 2: single-nucleotide polymorphism (SNP)-based heritability estimate for the second phenotype

§ Note: the heritability values derived from annotation stratified analyses may be sometimes negative, or very small. The noise in the estimation of single-nucleotide polymorphism (SNP)-based heritability may cause the genetic correlation estimates to be out of bounds (>1) or even be set as missing (NA) when the heritability estimates are small or below zero, respectively. In such cases, genetic covariance estimates are less biased than genetic correlation measures.

# **Table S11 (b).** Results of the genetic covariance analyses between schizophrenia and obesity stratified by insulin signalling gene-sets.

* Nominally significant stratified genetic covariance estimates (P < 0.05)

** Statistically significant stratified genetic covariance estimates (P < 2.06x10-4)

| *Gene-set name* | *n genes/gene-set* | *ρ_g_* | *SE ρ_g_* | *P* | *r_g_* | *h^2^_SNP_ 1* | *h^2^_SNP_ 2* | *annotated SNPs* | *total SNPs* |
| --- | --- | --- | --- | --- | --- | --- | --- | --- | --- |
| BIOCARTA INSULIN PATHWAY | 21 | -0.00048 | 0.00029 | 0.09793 | -0.51439 | 0.00161 | 0.00053 | 1,640 | 1,008,848 |
| KEGG INSULIN SIGNALING PATHWAY | 137 | -0.00066 | 0.00079 | 0.40342 | -0.19705 | 0.00553 | 0.00202 | 12,249 | 1,008,848 |
| PID INSULIN PATHWAY | 44 | -0.00069 | 0.00037 | 0.06077 | -0.56929 | 0.00146 | 0.00100 | 4,647 | 1,008,848 |
| REACTOME INSULIN PROCESSING | 27 | 0.00008 | 0.00019 | 0.68675 | § NA | 0.00087 | -0.00048 | 3,273 | 1,008,848 |
| REACTOME INSULIN RECEPTOR RECYCLING | 26 | 0.00080 | 0.00030 | 0.00877 * | § NA | 0.00297 | -0.00029 | 2,126 | 1,008,848 |
| REACTOME INSULIN RECEPTOR SIGNALLING CASCADE | 54 | -0.00076 | 0.00086 | 0.37614 | -0.19809 | 0.00424 | 0.00348 | 4,655 | 1,008,848 |
| REACTOME REGULATION OF INSULIN SECRETION | 77 | 0.00025 | 0.00100 | 0.80091 | 0.05869 | 0.01438 | 0.00129 | 10,322 | 1,008,848 |
| REACTOME SIGNALING BY INSULIN RECEPTOR | 78 | -0.00013 | 0.00105 | 0.90155 | -0.02627 | 0.00732 | 0.00333 | 6,595 | 1,008,848 |
| van de Vondervoort's gene-set | 53 | 0.00027 | 0.00077 | 0.72079 | § NA | 0.00529 | -0.00022 | 9,443 | 1,008,848 |

*Abbreviations*

ρ_g_: genetic covariance estimate

SE ρ_g_: standard error of the estimate of ρ_g_

P: p-value from the statistical test for genetic covariance

r_g_: genetic correlation estimate

h^2^_SNP_ 1: single-nucleotide polymorphism (SNP)-based heritability estimate for the first phenotype

h^2^_SNP_ 2: single-nucleotide polymorphism (SNP)-based heritability estimate for the second phenotype

§ Note: the heritability values derived from annotation stratified analyses may be sometimes negative, or very small. The noise in the estimation of single-nucleotide polymorphism (SNP)-based heritability may cause the genetic correlation estimates to be out of bounds (>1) or even be set as missing (NA) when the heritability estimates are small or below zero, respectively. In such cases, genetic covariance estimates are less biased than genetic correlation measures.

| **Table S11 (c).** Results of the genetic covariance analyses between schizophrenia and type 2 diabetes mellitus stratified by insulin signalling gene-sets. |
| --- |
| * Nominally significant stratified genetic covariance estimates (P < 0.05)  ** Statistically significant stratified genetic covariance estimates (P < 2.06x10-4) |

| *Gene-set name* | *n genes/gene-set* | *ρ_g_* | *SE ρg* | *P* | *r_g_* | *h^2^_SNP_ 1* | *h^2^_SNP_ 2* | *annotated SNPs* | *total SNPs* |
| --- | --- | --- | --- | --- | --- | --- | --- | --- | --- |
| BIOCARTA INSULIN PATHWAY | 21 | -0.00041 | 0.00018 | 0.02506 * | -0.43913 | 0.00160 | 0.00053 | 1,707 | 1,049,802 |
| KEGG INSULIN SIGNALING PATHWAY | 137 | 0.00080 | 0.00066 | 0.22494 | 0.20820 | 0.00551 | 0.00271 | 12,772 | 1,049,802 |
| PID INSULIN PATHWAY | 44 | -0.00058 | 0.00019 | 0.00248 * | -0.40806 | 0.00156 | 0.00128 | 4,833 | 1,049,802 |
| REACTOME INSULIN PROCESSING | 27 | 0.00006 | 0.00012 | 0.60493 | 0.05124 | 0.00088 | 0.00175 | 3,369 | 1,049,802 |
| REACTOME INSULIN RECEPTOR RECYCLING | 26 | -0.00008 | 0.00017 | 0.64100 | -0.03927 | 0.00298 | 0.00131 | 2,226 | 1,049,802 |
| REACTOME INSULIN RECEPTOR SIGNALLING CASCADE | 54 | -0.00052 | 0.00042 | 0.21107 | -0.17109 | 0.00418 | 0.00221 | 4,875 | 1,049,802 |
| REACTOME REGULATION OF INSULIN SECRETION | 77 | -0.00188 | 0.00079 | 0.01780 * | -0.30752 | 0.01478 | 0.00253 | 10,842 | 1,049,802 |
| REACTOME SIGNALING BY INSULIN RECEPTOR | 78 | -0.00059 | 0.00048 | 0.22190 | -0.12943 | 0.00727 | 0.00286 | 6,902 | 1,049,802 |
| van de Vondervoort's gene-set | 53 | -0.00024 | 0.00041 | 0.55948 | -0.05816 | 0.00597 | 0.00280 | 10,060 | 1,049,802 |

*Abbreviations*

ρ_g_: genetic covariance estimate

SE ρ_g_: standard error of the estimate of ρ_g_

P: p-value from the statistical test for genetic covariance

r_g_: genetic correlation estimate

h^2^_SNP_ 1: single-nucleotide polymorphism (SNP)-based heritability estimate for the first phenotype

h^2^_SNP_ 2: single-nucleotide polymorphism (SNP)-based heritability estimate for the second phenotype

§ Note: the heritability values derived from annotation stratified analyses may be sometimes negative, or very small. The noise in the estimation of single-nucleotide polymorphism (SNP)-based heritability may cause the genetic correlation estimates to be out of bounds (>1) or even be set as missing (NA) when the heritability estimates are small or below zero, respectively. In such cases, genetic covariance estimates are less biased than genetic correlation measures.
